# Plastid chaperone HSP90C guides precursor proteins to the SEC translocase for thylakoid transport

**DOI:** 10.1093/jxb/eraa399

**Published:** 2020-08-27

**Authors:** Tim Jiang, Bona Mu, Rongmin Zhao

**Affiliations:** 1 Department of Biological Sciences, University of Toronto Scarborough, Toronto, Ontario, Canada; 2 Department of Cell and Systems Biology, University of Toronto, Toronto, Ontario, Canada

**Keywords:** Chloroplast biogenesis, heat shock protein 90, molecular chaperone, photomorphogenesis, PSII, protein homeostasis, SEC translocon, thylakoid transport

## Abstract

Chloroplast stromal factors involved in regulating thylakoid protein targeting are poorly understood. We previously reported that in *Arabidopsis thaliana*, the stromal-localized chaperone HSP90C (plastid heat shock protein 90) interacted with the nuclear-encoded thylakoid lumen protein PsbO1 (PSII subunit O isoform 1) and suggested a role for HSP90C in aiding PsbO1 thylakoid targeting. Using *in organello* transport assays, particularly with model substrates naturally expressed in stroma, we showed that light, exogenous ATP, and HSP90C activity were required for Sec-dependent transport of green fluorescent protein (GFP) led by the PsbO1 thylakoid targeting sequence. Using a previously identified PsbO1T200A mutant, we provided evidence that a stronger interaction between HSP90C and PsbO1 better facilitated its stroma–thylakoid trafficking. We also demonstrated that SecY1, the channel protein of the thylakoid SEC translocase, specifically interacted with HSP90C *in vivo*. Inhibition of the chaperone ATPase activity suppressed the association of the PsbO1GFP–HSP90C complex with SecY1. Together with analyzing the expression and accumulation of a few other thylakoid proteins that utilize the SRP, TAT, or SEC translocation pathways, we propose a model in which HSP90C forms a guiding complex that interacts with thylakoid protein precursors and assists in their specific targeting to the thylakoid SEC translocon.

## Teaser

The chloroplast stromal chaperone HSP90C interacts directly with thylakoid SEC translocase to direct its client proteins for thylakoid transport in an ATPase activity-dependent manner.

## Introduction

Chloroplasts are essential plant organelles where photosynthesis takes place. They are a group of specialized plastids that are differentiated from proplastids or converted from other types of plastids (Jarvis and López-Juez, 2013). Development of chloroplasts depends on the import of thousands of nuclear-encoded proteins, which generally contain a special chloroplast-targeting peptide (cTP) at their N-termini (Petsalaki *et al.*, 2006). The cTPs are first recognized by cytosolic chaperones such as Hsp70 and Hsp90 (Lee *et al.*, 2018) before their binding to TOC receptor proteins Toc159 and Toc33/Toc34 (May and Soll, 2000; Qbadou *et al.*, 2006). Pre-proteins are then transported through TOC75, the β-barrel channel protein, into the intermembrane space, and subsequently across the TIC complex (Y.L. Chen *et al.*, 2016; O’Neil *et al.*, 2017; Ganesan *et al.*, 2018) with the aid of stromal chaperones such as heat shock proteins (HSPs) HSP93, cpHSP70, and HSP90C (Shi and Theg, 2010; Su and Li, 2010; Inoue *et al.*, 2013; Huang *et al.*, 2016), or a Ycf2–FtsHi complex (Kikuchi *et al.*, 2018).

Proteins destined for the thylakoid typically have a second transit peptide that is exposed after cleavage of the cTP (Richter and Lamppa, 1998). These specific thylakoid-targeting peptides (tTPs) lead the stromal intermediates/precursors towards one of the three conserved translocases at the thylakoid membrane (Schunemann, 2007; Albiniak *et al.*, 2012; Frain *et al.*, 2016). Thylakoid membrane proteins are integrated through the signal recognition particle (SRP)/ALBINO3 (ALB3) integrase (Moore *et al.*, 2000; Trösch *et al.*, 2015). Thylakoid lumen proteins are translocated by either the twin arginine translocation (TAT) translocase that transports folded proteins (Settles *et al.*, 1997; Mori *et al.*, 1999), or the SEC translocase for unfolded proteins (Yuan *et al.*, 1994; Mori *et al.*, 1999).

Chloroplast stromal factors involved in regulating thylakoid protein targeting are poorly understood. The HSP60 family chaperone CPN60 in Arabidopsis, known to bind and facilitate Rubisco folding and assembly (Rospert *et al.*, 1993), has been shown to aid in the proper thylakoid membrane insertion of Plsp1 via cpSecA1 and cpSecY1 (Endow *et al.*, 2015), and compromised CPN60 function affects post-germination seedling development (Sela *et al.*, 2020). Yet, less is known about the stromal factors within chloroplasts that directly or indirectly regulate protein targeting of SEC substrates destined for the thylakoid lumen. In *Escherichia coli*, chaperones such as SecB bind co- and/or post-translationally to unfolded SEC substrates and probably maintain them in molten globule-like states for delivery to the Sec translocon (Bechtluft *et al.*, 2007; Tsirigotaki *et al.*, 2017). Additionally, DnaK (Hsp70), DnaJ (Hsp40), and GroEL/ES (Hsp60/Hsp10) have been directly implicated in the Sec pathway through interaction with SecB (Phillips and Silhavy, 1990; Sakr *et al.*, 2010; Calloni *et al.*, 2012; Castanie-Cornet *et al*., 2014). In the plant Arabidopsis, we recently identified a transient interaction between HSP90C and PsbO1, a nuclear-encoded thylakoid lumen subunit of the PSII complex (Jiang *et al.*, 2017). We showed that inhibiting HSP90C activity altered the distribution of the PsbO1–green fluorescent protein (GFP) fusion protein between the stroma and thylakoid compartments. While PsbO1 appears to be a model substrate for HSP90C, the exact role of HSP90C in the SEC translocation pathway is unclear.

HSP90C is an essential stromal chaperone for chloroplast biogenesis and thylakoid maturation (Ticha *et al*., 2020) and is also termed CR88 (Cao *et al.*, 2000, 2003). In green algae, this chaperone forms a complex consisting of HSP70B, CDJ1 (homolog of HSP40), and CGE1 (homolog of GrpE-type nucleotide exchange factor of bacterial HSP70s) (Schroda and Muhlhaus, 2009) similar to the cytosolic HSP90 complex. HSP90C has been shown to interact with components of the TOC–TIC, complex, and its ATPase activity has been implicated in chloroplast envelope protein import alongside HSP93 (Inoue *et al.*, 2013). As a molecular chaperone, HSP90C has been shown to regulate the function of other proteins such as PsbO1 (Jiang *et al.*, 2017) and VIPP1 post-translationally (Feng *et al.*, 2014). On the other hand, the chaperone’s own expression and accumulation are also developmentally regulated, and transgene-induced silencing caused impaired chloroplast development (Oh *et al.*, 2014) as well as accumulation of reactive oxygen species (Islam *et al.*, 2020). Addtionally, HSP90C expression is regulated by the ubiquitin–proteasome system in the cytoplasm and degraded even before import into plastids (Zhang *et al.*, 2019).

In this study, we aim to understand how HSP90C regulates thylakoid transport of SEC substrates into the thylakoid lumen. We isolated chloroplasts from transgenic plants containing stroma-accumulated GFP that was fused to the tTP of PsbO1, and then conducted an *in organello* chase assay. We found that HSP90C and its ATPase activity were required for the thylakoid translocation of SEC-dependent PsbO1 substrates. Using a previously identified PsbO1T200A mutant, we also provided evidence that a stronger interaction between HSP90C and PsbO1 better facilitated its thylakoid transport. Characterization of *de novo* synthesized, thylakoid-targeted proteins during photomorphogenesis suggests that HSP90C probably affected the stromal trafficking and subsequent thylakoid accumulation of substrates of the SEC pathway, while having little effect on the TAT and SRP translocase system. In addition, we showed that HSP90C interacted with the channel protein SecY1 using bimolecular fluorescence complementation (BiFC) and *in vivo* co-immunoprecipitation (co-IP). To our knowledge, this is the first report showing that the stromal molecular chaperone HSP90C associates with the thylakoid SEC translocase and aids in SEC substrate transport in an ATPase-dependent manner.

## Materials and methods

### Plant materials and growth conditions

The *A. thaliana* ecotype Columbia (Col-0) was used as the wild type. To grow seedlings *in vitro*, seeds were surface sterilized and sown on half-strength Murashige and Skoog (MS) medium. After stratification in the dark at 4 °C for 3–4 d, seeds were cultured within a plant growth incubator set at 120 μmol m^−2^ s^−1^, 16/8 h light/dark cycle at 22 °C. Transgenic seedlings used were generated previously (Oh *et al.*, 2014; Jiang *et al.*, 2017). The T7-tagged SecY1 (SCY1) line was previously described (Skalitzky *et al.*, 2011) and was kindly provided by Dr Donna Fernandez (University of Wisconsin, USA).

### Transient protein expression analysis and bimolecular fluorescence complementation

Coding regions of PsbO^1–58^, PsbO1, HSP90C, SecY1, and RbcS3B were amplified using the primers listed in Supplementary Table S1 at *JXB* online, cloned into the pENTR207 vector by the Gateway system (Invitrogen), and then the BiFC destination vectors pB7WGYN9 or pB7WGYC9 containing nYFP/cYFP (yellow fluorescent protein) at their C-termini were prepared as previously described (Tsuda *et al.*, 2017). Plasmids used to overexpress PsbO1^1–58^GFP, PsbO1^1–85^GFP, PsbO1^1–332^GFP, PsbO1^1–332^YFP, HSP90C-FLAG, and PsbO1^1-332^GFP with HSP90C-FLAG overexpressed were generated as described previously (Oh *et al.*, 2014; Jiang *et al.*, 2017) and are listed in Supplementary Table S2. Plasmids for BiFC and transient expression analysis were transformed into *Agrobacterium tumefaciens* strain GV2260. *Nicotiana benthamiana* plants were grown for 4 weeks under long-day conditions (16 h of light, 8 h of darkness) at 28 °C. Leaves were syringe-infiltrated as previously described (Lewis *et al.*, 2008). Full-length constructs were infiltrated at a final OD_600_ of 0.5, and the BiFC construct was infiltrated at a final OD_600_ of 0.2, and plants were imaged after 48 h by confocal microscopy as previously described (Duong *et al.*, 2017). Whenever needed, 10 mM sodium azide and/or 30 μM geldanamycin were used to treat mesophyll cells after peeling the lower epidermis before monitoring the fluorescence.

### Co-immunoprecipitation analysis

To purify Myc-tagged complexes, co-IP of chloroplast membrane complexes was performed as previously described (Y.E. Chen *et al.*, 2016; Kikuchi *et al.*, 2018). Isolated intact tobacco chloroplast was resuspended to 1.0 mg chlorophyll ml^–1^. An equal volume of solubilization buffer [2% digitonin, 100 mM Tris–HCl, pH 7.5, 20% (w/v) glycerol, 300 mM NaCl, 5 mM EDTA, 5 mM MgCl_2_, one tablet of Roche protease inhibitor in 10 ml, and 20 μM chymostatin] was then added and the membranes were solubilized in the dark on ice for 10 min. Insoluble material was removed through centrifugation at 18 000 *g* for 20 min. The resulting supernatant was incubated with protein A–agarose and anti-Myc antibody for 2 h in a cold room. After washing four times, the bound proteins were eluted with SDS buffer and analyzed by immunoblotting. To purify the T7-tagged SecY1 complex from Arabidopsis seedlings, a similar procedure was used, except that a final concentration of 0.5% DDM (*n***-**dodecyl β-d-maltoside) was used instead of digitonin to solubilize the chloroplast membranes, and anti-T7 agarose was used to capture the T7-tagged SecY1 complex in Arabidopsis, in the absence or presence of 10 mM sodium azide or 30 μM geldanamycin.

### Fluorescence and confocal microscopy

Fluorescence microscopy was performed using an upright Zeiss LSM 510 confocal laser scanning microscope (Carl Zeiss). The excitation/emission wavelengths were used as follows: for GFP, 488 nm/500–530 nm; for cyan fluorescent protein (CFP), 440 nm/460–490 nm; for YFP, 514 nm/525–552 nm; and for chlorophyll, 633 nm/650–720 nm. To avoid overlap between the fluorescence channels, sequential *z*-stack scanning was used when necessary. Images were processed by ImageJ (National Institutes of Health) or ZEN 2.3 (Carl Zeiss Microscopy GmbH, 2011).

### Chloroplast isolation, lysis, and fractionation

Intact chloroplasts were isolated from 10- to 12-day-old seedlings and quantification was done as previously described (Jiang *et al.*, 2017). Chloroplasts were resuspended in HMS buffer (330 mM sorbitol, 50 mM HEPES-NaOH pH 8.0, 3 mM MgSO_4_) at 1 mg ml^–1^ chlorophyll. Chloroplasts for fractionation analysis were resuspended in osmotic lysis buffer (20 mM HEPES-KOH pH 8.0, 10 mM NaHCO_3_, 2 mM MgCl_2_, 2.5 mM EDTA, 2.5 mM EGTA, one tablet of Roche protease inhibitor in 20 ml, and 10 μM chymostatin) on ice for 15 min. Supernatant (stroma) and pellet (thylakoid) fractions were separated with centrifugation at 2600 *g* for 5 min. The pellet fraction was washed three times in HMS buffer. Stroma proteins were concentrated using trichloroacetic acid (TCA) precipitation (20%) on ice for 10 min followed by centrifugation at 13 000 *g* for 30 min at 4 °C. The pellet was rinsed with 200 μl of ice-cold acetone and centrifuged for 10 min at 4 °C. This step was repeated, and the pellet was air dried. Both the membrane pellet and precipitated supernatant pellet were dissolved in stoichiometrically equivalent volumes of 2× SDS sample buffer with 5 M urea before being subjected to immunoblotting analysis. To confirm thylakoid lumen-located proteins, fractionated thylakoid fractions were further treated with thermolysin or trypsin according to previously described procedures (Endow *et al.*, 2015). Briefly, the thylakoid pellet was washed twice with HS (50 mM HEPES-KOH, pH 8.0, 330 mM sorbitol) buffer and resuspended in 1× HS buffer containing thermolysin (0.1 mg ml^–1^, Sigma, P1512) and 10 mM CaCl_2_ or trypsin (60 μg ml^–1^, Sigma, T5266), and incubated on ice in the dark for 20 min. The protease treatment was quenched with an equal volume of HS buffer containing 20 mM EDTA. The sample was spun-down and subjected to SDS–PAGE analysis.

### *In organello* thylakoid transport assay

Intact chloroplasts from 10 g of 12-day-old PsbO1^1–85^GFP seedlings were isolated and resuspended in HMS buffer containing 10 μM chymostatin (BioShop) to inhibit serine proteases. Chloroplasts (0.2 mg ml^–1^ chlorophyll) were incubated at 25 °C under 120 μmol m^−2^ s^−1^ white light or in the dark (wrapped by aluminum foil) for 12–60 min. Transport in the dark was induced by adding 5 mM Mg-ATP (BioShop) to intact chloroplasts. Reactions were stopped using HMS buffer containing EDTA to a final concentration of 10 mM. Alternatively, transport assays were conducted in the presence of 10 mM sodium azide, 10 μM geldanamycin (Sigma, G3381), 10 μM 3-(3,4-dichloro-phenyl)-1,1-dimethylurea (DCMU; Sigma-Aldrich), or 12 μM 2,5-dibromo-6-isopropyl-3-methyl-1,4-benzoquinone (DBMIB; Sigma-Aldrich). DCMU and DBMIB were prepared as 1000-fold concentrated stocks in 95% ethanol prior to dilution in H_2_O just prior to use.

### Antibodies

Polyclonal rabbit anti-HSP90C and anti-PsbO1 antibodies were previously described (Jiang *et al.*, 2017). Other primary antibodies used in this study include anti-GFP polyclonal antibody (Sigma, G1544), anti-FLAG monoclonal antibody (Sigma, F3165), anti-LHCB2 (Agrisera, AS01 003), anti-PsbS (Agrisera, AS09 533), anti-PsbA (Agrisera, AS05 084), anti-PC (Agrisera, AS06 141), anti-PsbP (Agrisera, AS06 142-23), anti-ClpC (AS01001), anti-CPN60A1 (AS122613), anti-Myc (ThermoFisher, AHO0062), and anti-HA antibodies (Roche, 11 867 423 001). GFP-TRAP resin is from ChromoTek. Anti-T7 antibody agarose was from Abcam (ab1230). Protein A–agarose was purchased from Sigma (P1406).

## Results

### Inhibition of HSP90C ATPase activity reduces thylakoid SEC translocation efficiency

**Fig. 1.**
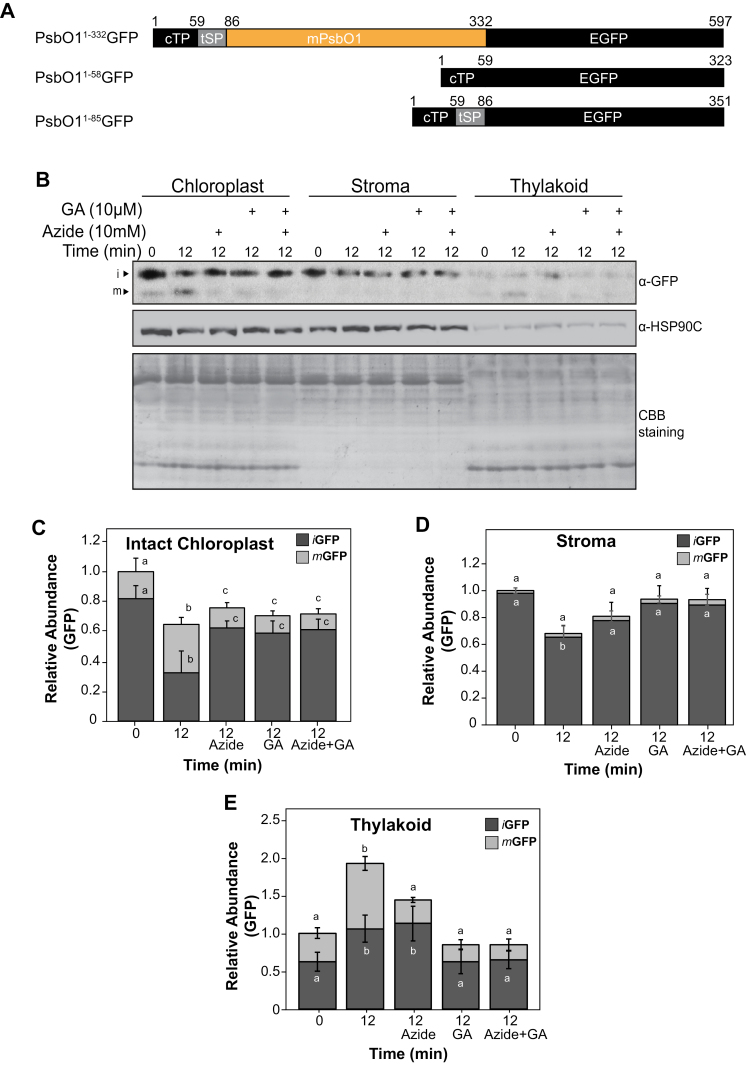
Inhibition of HSP90C activity reduced SEC-dependent thylakoid protein transport in isolated chloroplasts. Intact chloroplasts from PsbO1^1–85^GFP-expressing seedlings were used to chase the thylakoid transport of GFP protein under illumination with 110 μmol m^−2^ s^−1^ at 22 °C. (A) Schematic diagram of expressed fusion proteins. All three constructs are based on the pEGAD vector, and the 27 amino acid linker region after the GFP C-terminus in each construct is not shown. (B) Analysis of GFP and fusion proteins from total chloroplast lysates or fractionated stroma and thylakoid fractions. Sodium azide (Azide) and/or geldanamycin (GA) were included in the chase period under light. (C–E) Relative amounts of stromal intermediate and thylakoid mature GFP signals. The total GFP signals at time 0 min for intact chloroplast, stroma, and thylakoid fractions were used as references, respectively, and treated as 1.0. Error bars represent the SD from four independent assays, and significant levels for *i*GFP and *m*GFP were tested independently. The same letters beside the error bars show that the differences are not statistically significant (*P*>0.05).

We have previously reported that the Arabidopsis chloroplast stroma chaperone HSP90C interacts with PsbO1, a PSII subunit that is naturally located in the thylakoid lumen (Jiang *et al.*, 2017). By analyzing the expression and chloroplast targeting of PsbO1–GFP fusion protein, we showed in our previous study that altered HSP90C activity affected the intermediate and mature PsbO1–GFP fusion protein distribution between the stroma and thylakoid compartments. However, that study was based on analyzing the steady-state accumulation of PsbO1 fusion proteins in whole-plant lysates and did not show how the fusion proteins crossed the thylakoid membrane via the SEC translocon (Yuan *et al.*, 1994). To understand how HSP90C is involved in the active thylakoid transport of PsbO1, we isolated chloroplasts from lines expressing different reporter proteins (Fig. 1A), and monitored the entry of stromal-localized intermediate GFP (*i*GFP) fusion proteins, led by the PsbO1 thylakoid-targeting sequence, into the thylakoid lumen using *in organello* assays. These isolated chloroplasts were enriched with *i*GFP which was confirmed by chloroplast isolation and fractionation (Supplementary Fig. S1A). The lumen localization was further verified following a protease protection assay using trypsin and thermolysin (Supplementary Fig. S1B). The PsbO1^1–85^GFP line was chosen for further analysis rather than the full-length PsbO1^1–332^GFP line since it still maintains interaction with HSP90C while having a less severe effect on the overall chloroplast maturation, and was less prone to aggregation (Jiang *et al.*, 2017). From the *in organello* assays under light, it was clear that a significant amount of *i*GFP was reduced while mature GFP (*m*GFP) significantly increased after 12 min (Fig. 1B–E; Supplementary Fig. S2A), suggesting successful thylakoid transport. To understand the role of HSP90C and the SEC translocon in active PsbO1 fusion protein transport, sodium azide, a chemical commonly used to inhibit SEC transport activity (Yuan and Cline, 1994), and geldanamycin, a specific HSP90 ATPase inhibitor (Roe *et al.*, 1999), were included in the assays. Immunoblotting and quantitative analyses on either total chloroplast lysates (Fig. 1C) or fractionated stroma and thylakoid fractions (Fig. 1D, E) all showed that geldanamycin and azide similarly suppressed thylakoid *m*GFP accumulation. This indicates that HSP90C activity is required for active SEC-dependent transport of GFP led by the PsbO1 thylakoid-targeting sequence.

**Fig. 2.**
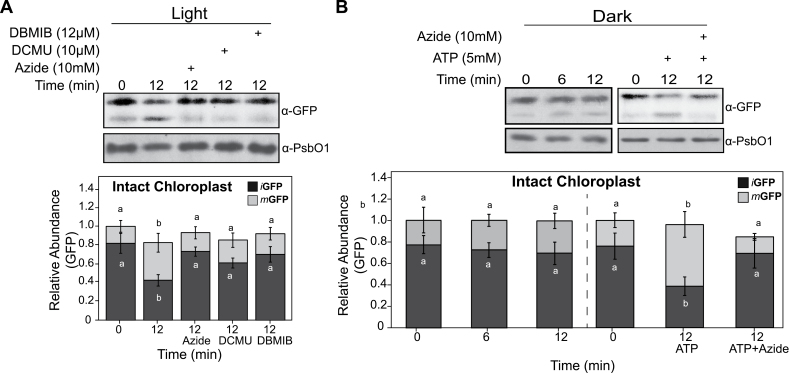
Thylakoid transport requires energy from photosynthesis or exogenous ATP, and is inhibited by sodium azide. Intact chloroplasts from PsbO1^1–85^GFP-expressing seedlings were used to chase the thylakoid transport of GFP protein under light with or without the photosynthetic inhibitors DBMIB, DCMU, or sodium azide (A), or in the dark with exogenous ATP or sodium azide (B). Immunoblottings with anti-GFP and anti-PsbO1 were performed. Quantitative analyses of both stromal GFP (*i*GFP) and thylakoid GFP (*m*GFP) are shown below the blots. The total GFP signals at time 0 min for intact chloroplast, stroma, and thylakoid fractions were used as references, respectively, and treated as 1.0. Error bars represent the SD from independent assays, and significant levels for *i*GFP and *m*GFP were tested independently. The same letters beside the error bars show that the differences are not statistically significant (*P*>0.05).

The SEC translocase requires ATP to drive protein transport. Treatment of isolated chloroplasts with the photosynthesis inhibitors DCMU or DBMIB that decrease the intracellular ATP level, suppressed the transport of *i*GFP (Fig. 2A). We also expected that, when conducted in the dark, the *in organello* assays would result in reduced transport of the SEC substrates. Indeed, active transport of *i*GFP in the dark was only visible when exogenous ATP was added (Fig. 2B). Similarly, the thylakoid lumen localization of the transported GFP was confirmed by treating the thylakoid fractions with thermolysin (Supplementary Fig. S2B). Together, these data indicate that HSP90C may act as an adaptor within the stroma and play active roles in facilitating SEC transport of intermediate PsbO1 (*i*PsbO1) into the thylakoid lumen.

### Stronger interaction between HSP90C and PsbO1 better facilitates thylakoid localization

**Fig. 3.**
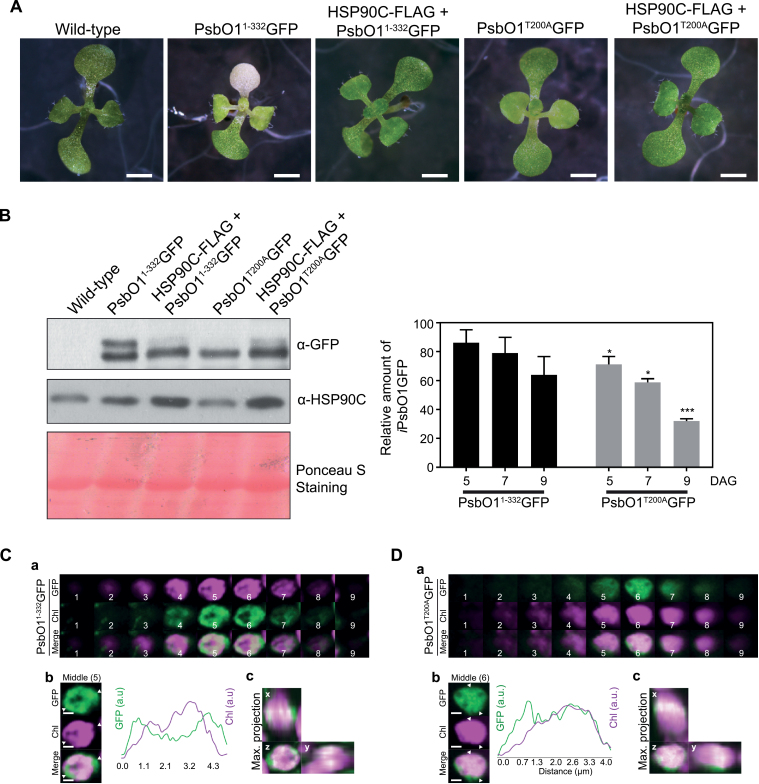
Seedlings expressing PsbO1^T200A^GFP showed a phenotype similar to those co-expressing PsbO1^1–332^GFP and HSP90C-FLAG. (A) Eleven-day-old seedlings grown on MS medium at 22 °C, 110 μmol m^−2^ s^−1^ under a 16 h light/8 h dark photoperiod. Scale bars represent 2.5 mm. (B) Representative immunoblots of samples made from seedlings shown in (A) with anti-GFP and anti-HSP90C antibodies (left). Quantitative analysis of stromal intermediate forms of PsbO1^1–332^GFP and PsbO1^T200A^GFP relative to total GFP fusion proteins in seedlings grown at 5, 7, and 9 d old. Four independent lines were used. *, **, and *** represent statistically significant differences (*P*<0.05, 0.01, and 0.005, respectively) compared with those in PsbO1^1–332^GFP line at the same age. (C and D) Chloroplasts of mesophyll cells in seedlings at 4 d after germination (DAG) expressing PsbO1^1–332^GFP (C) and PsbO1^T200A^GFP (D). GFP and chlorophyll autofluorescence channels are displayed as individual *z*-stacks and a composite image (a). Fluorescence intensity profiles of GFP and chlorophyll (Chl) were analyzed using the representative middle slice (fifth in C and sixth in D) (b). The maximum intensity projection of the *z-*axis, *x*-axis, and *y*-axis is also shown (c). Scale bar=1μm.

Previously, we identified a PsbO1^T200A^ mutant, and this mutant protein interacted more strongly with HSP90C (Jiang *et al.*, 2017). The mutant protein also has a larger Stokes radius compared with the wild type, and may form oligomers or adopt a more disordered structure. Thr200 resides in a highly conserved region (Supplementary Fig. S3) and the mutation may significantly disrupt the partially folded PsbO1 structure. To investigate how the point mutation in PsbO1 might affect its suborganellar trafficking, we fused GFP to the C-terminal end and generated stable Arabidopsis lines expressing PsbO1^T200A^GFP. Expression of PsbO1^1–332^GFP caused a severe variegation phenotype as previously observed; surprisingly, expression of PsbO1^T200A^GFP led to a diminished variegation phenotype in all four independent transgenic lines (Fig. 3A). We also observed greater accumulation of the mature form during early development stages, similar to the effect of co-expressing HSP90C-FLAG with PsbO1^1–332^GFP (Fig. 3B). This mature PsbO1^T200A^GFP protein was enriched within the thylakoid lumen, as fluorescence microscopy analysis showed enhanced GFP co-localization with chlorophyll (Fig. 3C, D). This suggests that a stronger interaction between PsbO1^T200A^ and HSP90C may better facilitate its thylakoid transport.

### Accumulation of endogenous thylakoid SEC substrates during photomorphogenesis was delayed with overloading of artificial SEC substrates in the stroma

Many of the proteins necessary for photosynthesis are synthesized as pre-proteins within the cytosol, targeted for chloroplast stroma across the chloroplast envelopes, then inserted into or transported through the thylakoid membrane. We have previously shown that constitutive expression and accumulation of *i*PsbO1^59–332^GFP in stroma acts as a proteotoxic stressor (Jiang *et al.*, 2017). To better understand how the presence of artificial SEC substrates such as PsbO1^1–332^GFP or PsbO1^1–85^GFP impacted the trafficking of endogenous thylakoid proteins, we analyzed the accumulation of a few PSII proteins during photomorphogenesis. Photomorphogenesis of young seedlings was monitored for up to 48 h (Supplementary Fig. S4A). We then analyzed the accumulation of nuclear-encoded thylakoid proteins PsbO, PsbS, and LHCB2, and a plastid-encoded PsbA, all related to PSII and important for proper thylakoid biogenesis and function (Armarego-Marriott *et al.*, 2019). PsbS and LHCB2 are integrated into the thylakoid membrane spontaneously or through the SRP pathway, whereas PsbA requires both SRP components and SEC components (Walter *et al.*, 2015; Hristou *et al.*, 2019). Compared with the wild type or seedlings expressing HSP90C-FLAG or PsbO1^1–58^GFP, seedlings expressing PsbO1^1–85^GFP and PsbO1^1–332^GFP accumulated mature PsbO1 (*m*PsbO1) and PsbA much more slowly, while mature PsbS and LHCB2 followed a similar wild-type-like trend during the photomorphogenesis period (Supplementary Fig. S4B–D, F–I). As was previously observed (Oh *et al.*, 2014), the relative level of steady-state HSP90C abundance appeared to be decreasing over time, probably due to increases in expression of light-activated photosynthetic proteins, thereby causing HSP90C to make up less of the total percentage of plastid proteins (Supplementary Fig. S4E). Interestingly, in PsbO1^1–85^GFP and PsbO1^1–332^GFP seedlings, more HSP90C was observed during the early photomorphogenic period (0–4 h and 0–8 h, respectively) prior to decreasing (Supplementary Fig. S4E). Introducing more HSP90C-FLAG enhanced the absolute level of HSP90C after the onset of photomorphogenesis; however, the overall expression pattern still followed a wild-type-like trend (Supplementary Fig. S4E), possibly through other negative feedback regulations (Rutgers *et al*., 2017). To understand whether the other chaperones are affected during photomorphogenesis, we analyzed CLPC, a HSP100 family unfoldase (Flores-Perez et al., 2016), chloroplast HSP70 which we previously showed to be enriched with PsbO1^1–332^GFP expression (Jiang *et al.*, 2017), and CPN60A, a subunit of the HSP60 chaperonin that is well documented as being involved in RbcL maturation and PlsP1 thylakoid targeting (Endow *et al.*, 2015). Interestingly, the expression pattern of HSP70 over time is very similar to that of HSP90C and their decreases over time are both slower in PsbO^1–85^GFP compared with the wild-type line (Supplementary Fig. S5). We also noticed that CPN60A and CLPC expression did not decrease as much as HSP90C and HSP70 during photomorphogenesis; however, we did not see a dramatic difference for these two proteins between the wild type and the other three lines expressing PsbO1GFP fusion proteins (Supplementary Fig. S5). This suggests that HSP90C, probably with HSP70, plays a more specific role in regulating PsbO1 fusion protein transport via SEC translocase.

**Fig. 4.**
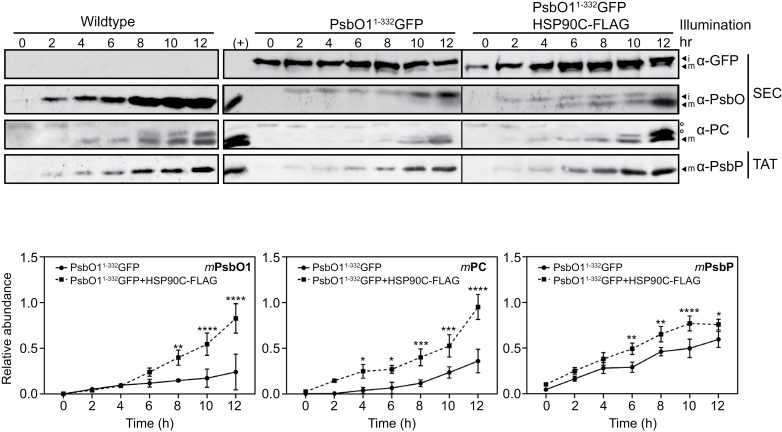
HSP90C aids in the SEC-dependent protein transport into the thylakoid during the early stage of photomorphogenesis. Seeds from the wild type and PsbO1^1–332^GFP- and PsbO1^1–332^GFP-co-expressing HSP90C-FLAG lines were stratified and grown at 22 °C in complete darkness for 3.5 d and then switched to constant light at 110 μmol m^−2^ s^−1^ for 48 h. Seedlings collected at different time points were directly ground in Laemmli buffer and loaded based on an equal number of seedlings. Anti-GFP, PsbO, PC, and PsbP antibodies were used for immunoblotting (top). Mature PsbO1 (*m*PsbO1), PC1 (*m*PC), and PsbP (*m*PsbP) signals were quantitated and normalized to the positive control (+) samples, and are shown at the bottom. The positive control (+) for all samples comes from wild-type seedlings at 4 DAG grown under a 16 h light/8 h dark/light cycle. It should be noted that anti-PC antibody blotting normally yields multiple bands that may represent differently charged states, or processing forms of PC. We labeled the bottom band as the mature form and quantitated its amount relative to all band signals. Error bars represent the SD for three biological assays. *, **, ***, and **** represent significantly different levels of the protein in the PsbO1^1–332^GFP+HSP90C-FLAG line compared with the PsbO1^1–332^GFP line at *P*<0.05, 0.01, 0.005, and 0.001, respectively.

Given that the major difference in HSP90C expression between the wild type and the other lines occurred within the first 12 h (Supplementary Fig. S4, S5), we analyzed the dynamics of SEC function *in vivo* during this period of photomorphogenesis by measuring the relative amounts of both *i*/*m*PsbO1^1–332^GFP and endogenous *i*/*m*PsbO1. We found that in PsbO1^1–332^GFP transgenic seedlings, endogenous *m*PsbO1 accumulated faster during the de-etiolation process when there was co-expression of HSP90C-FLAG (Fig. 4). We also analyzed the levels of another SEC substrate, plastocyanin PC1, and the TAT substrate PsbP. PC1 showed a trend similar to PsbO1 in that we observed less mature and larger forms, either the pre-protein or the stromal intermediate given the decreasing patterns at early stages, whereas PsbP was only observed in its mature form (Fig. 4). A delay in SEC-dependent thylakoid trafficking but not chloroplast import should result in the stable accumulation of the endogenous stromal forms, as was observed for PsbO1 and PC1, but not for the TAT substrate *i*PsbP (Fig. 4). Together, these results suggest that there might be a limited capacity for the stable accumulation of SEC substrates within the stroma during the early chloroplast development stage.

### Transient expression of PsbO1^1–332^GFP in tobacco mesophyll chloroplasts results in formation of punctae

**Fig. 5.**
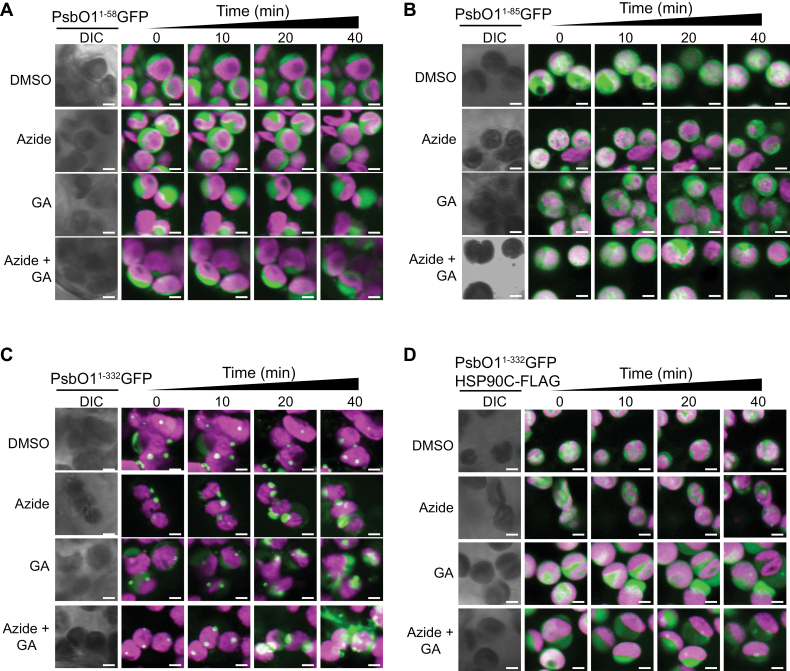
Transient expression of PsbO1^1–332^GFP fusion proteins in tobacco leaf mesophyll chloroplasts. (A–D) Laser scanning microscopy images were taken from mesophyll cells of *N. benthamiana* transiently expressing GFP that was fused to PsbO1 chloroplast targeting sequences (A, B), PsbO1 (C), or PsbO1 and co-expressing HSP90C-FLAG (D). Tobacco mesophyll chloroplasts were treated with 10 mM sodium azide (Azide) and/or 30 μM geldanamycin (GA) at 3 d after infiltration, and monitored for 40 min at 10 min intervals. Scale bars represent 2.5 µm. Only merged images for GFP signals (green) and chlorophyll (magenta) are shown.

To further understand the specific role of HSP90C in mediating PsbO1 transport across the thylakoid membrane and/or PsbO1 homeostasis within chloroplasts, GFP fusion proteins led by different chloroplast- and thylakoid-targeting sequences (Fig. 1A) were transiently expressed in tobacco *N. benthamiana* leaf mesophyll chloroplasts after infiltration with *A. tumefaciens* containing corresponding binary plasmids. PsbO1^1–58^GFP-expressing chloroplasts had GFP signals localized primarily to chloroplast stroma, and sodium azide and/or geldanamycin did not affect the GFP fluorescence distribution (Fig. 5A). PsbO1^1–85^GFP-expressing chloroplasts had GFP signals localized to both the stroma and thylakoid, as represented by increased overlapping of the GFP signal with chlorophyll fluorescence, and treatment with sodium azide and/or geldanamycin reduced the thylakoid association of GFP signals (Fig. 5B). To confirm that GFP was located in the thylakoid lumen in PsbO1^1–85^GFP-expressing chloroplasts, we isolated chloroplasts from infiltrated tobacco leaves treated or not with geldanamycin and azide, and then fractionated the chloroplasts into stroma and thylakoid fractions. Immunoblotting analysis confirmed the presence of mature GFP-sized proteins in the thylakoid fraction and the inhibitory effects of geldanamycin and azide (Supplementary Fig. S6). Surprisingly, we observed punctae of signal in PsbO1^1–332^GFP-expressing tobacco chloroplasts, which were enlarged upon treatment with azide or geldanamycin (Fig. 5C). These punctae were reminiscent of those previously observed in Arabidopsis chloroplasts in lines stably transformed with PsbO1^59–332^GFP (*i*PsbO1GFP), which was shown to form aggregates (Jiang *et al.*, 2017). Transient co-expression of HSP90C with PsbO1^1–332^GFP did not result in any observable punctae unless azide and/or geldanamycin were applied (Fig. 5D).

To further understand the exact subplastidic localization of these punctae, we transiently co-expressed RbcS^1–79^CFP with PsbO1^1–332^YFP. Due to partial overlap between YFP and CFP signals, the strong signal from PsbO1^1–332^YFP punctae leaked over to the CFP channel (Supplementary Fig. S7A). Nevertheless, the presence of CFP within the stroma allowed us to clearly define the peripheral border of the stroma (Supplementary Fig. S7B). *Z*-stack analysis indicated that the majority of punctae fell within two categories, one co-localized with the thylakoid membrane and the other near the periphery of the stroma where the inner chloroplast envelope and thylakoid may be joined (Supplementary Fig. S7C, D). It was possible that the punctae were formed from the stromal PsbO1^1–322^YFP in proximity to the SEC or TIC translocon due to insufficient chaperone activity at these specific sites.

### HSP90C interacts with SecY1 and facilitates substrate association with the thylakoid

**Fig. 6.**
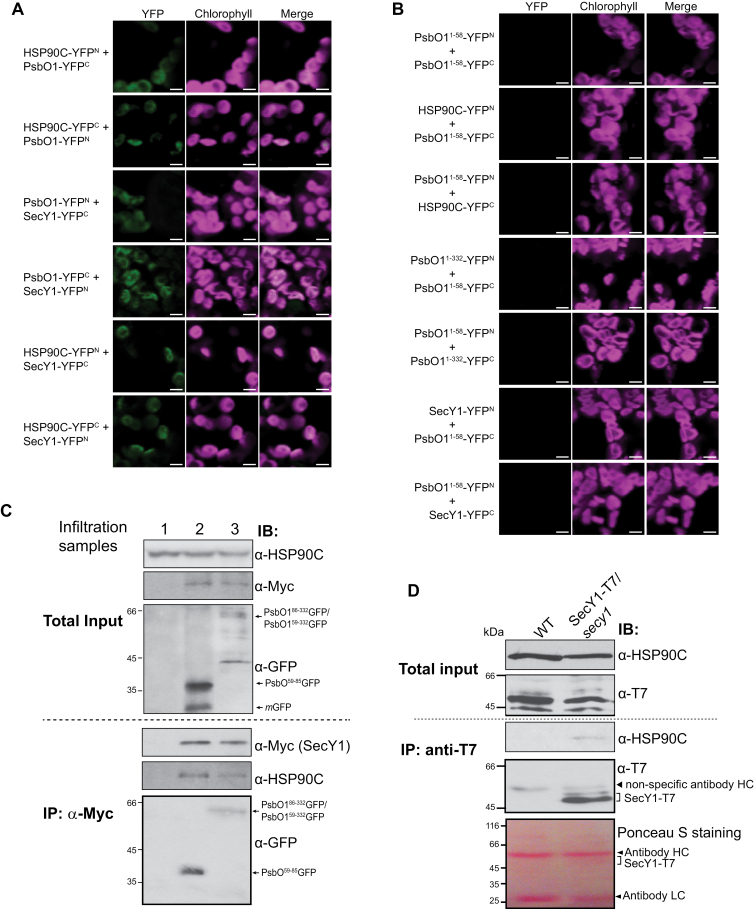
Split YFP-based bimolecular fluorescence complementation and co-IP analyses. (A and B) Laser scanning microscopy images were taken from mesophyll cells of *N. benthamiana* transiently co-expressing YFP N- or C-half fusion proteins that are labeled as -YFP^N^ and -YFP^C^ respectively. Fluorescence images for YFP, chlorophyll, and the merged signals are shown. Scale bars represent 5 µm. (C) Co-IP of the Arabidopsis SecY1 complex from tobacco leaves after co-infiltration and transient expression of YFP^N^-tagged SecY1 and GFP fusion proteins that were led by PsbO1^1–85^ (lane 2) or full-length PsbO1 (PsbO1^1–332^) (lane 3). Lane 1 is the wild-type control. YFP^N^ was also Myc tagged. Anti-HSP90C antibody recognizes both Arabidopsis and tobacco chloroplast HSP90C. It should be noted that all full-length GFP fusion constructs are based on the pEGAD vector and there is a 27 amino acid linker region after the GFP C-terminus yielding a slightly larger GFP fusion protein than normally expected. Construct details are also shown in Fig. 1A. (D) Co-IP of the SecY1 complex from 12-day-old SECY1–T7/*secy1* Arabidopsis seedlings using anti-T7 antibody agarose. Wild-type (WT) seedlings were used as a negative control. Anti-HSP90C and anti-T7 antibodies were used to detect HSP90C and SecY1 proteins in both total input and the pull-down (IP) samples.

We have now shown that HSP90C activity affected SEC substrate protein translocation and stability within the stroma. It is possible that HSP90C interacts directly with the SEC translocase. Given that the BiFC assay is often used to visualize protein interaction between subunits of different protein complexes such as Toc75 of the TOC complex with Tic22 of the TIC complex (Y.L. Chen *et al.*, 2016), we tested whether HSP90C interacts with the SEC translocon *in vivo* using BiFC in *N. benthamiana*. As expected, a strong YFP fluorescence signal was observed when PsbO1 and HSP90C, or PsbO1 and SecY1, were paired (Fig. 6A), thus confirming that HSP90C interacted with PsbO1, and PsbO1 interacted with SecY1 within chloroplasts as previously reported (Yuan *et al.*, 1994; Schuenemann *et al.*, 1999; Jiang *et al.*, 2017). We also observed strong signal for the pair HSP90C and SecY1, whereas no signal was visible when either half of the YFP protein alone (Fig. 6B) was targeted to the chloroplast using PsbO1^1–58^, though they were well expressed in tobacco leaves (Supplementary Fig. S8), or when full-length RbcS was fused to either half of the YFP protein (Supplementary Fig. S9), suggesting that HSP90C specifically interacted with SecY1 *in vivo*.

To confirm the BiFC results, we infiltrated tobacco leaves with *Agrobacterium* expressing SecY1–nYFP together with PsbO1^1–332^GFP or PsbO1^1–85^GFP, and performed co-IP analysis with anti-Myc antibody, as the SecY1–nYFP was also Myc tagged. Endogenous tobacco HSP90C, Arabidopsis PsbO1^59–332^GFP, and PsbO1^86–332^GFP fusion protein were co-purified with Arabidopsis SecY1 protein (Fig. 6C). When the PsbO1^1–85^GFP construct was co-infiltrated, we also detected the intermediate form, PsbO1^59–85^GFP, which was derived from PsbO1^1–85^GFP after cleavage of the cTP (Fig. 6C). This result suggests that the co-IP analysis was specific as the tobacco HSP90C was not detected in control samples, neither were the partially degraded GFP fusion proteins, though they were clearly visible in the total input. To further confirm SecY1 and HSP90C interaction within Arabidopsis chloroplasts, we took advantage of an Arabidopsis line that had the endogenous *SECY1* gene knocked out, but with a T7 epitope-tagged *SECY1* under its native promoter (Skalitzky *et al.*, 2011). It should be clarified that under normal immunoblot conditions, SecY1–T7 does not appear to be well distinguished from non-specific signal due either to low binding sensitivity of the T7 antibody or to a low steady-state level of the protein (Fig. 6D). However, by using T7-affinity resin, we were able to purify a significant amount of SecY1–T7 from intact chloroplasts in which we identified co-enrichment of HSP90C (Fig. 6D), suggesting that HSP90C was indeed associated with the Arabidopsis chloroplast SEC translocase *in vivo*.

**Fig. 7.**
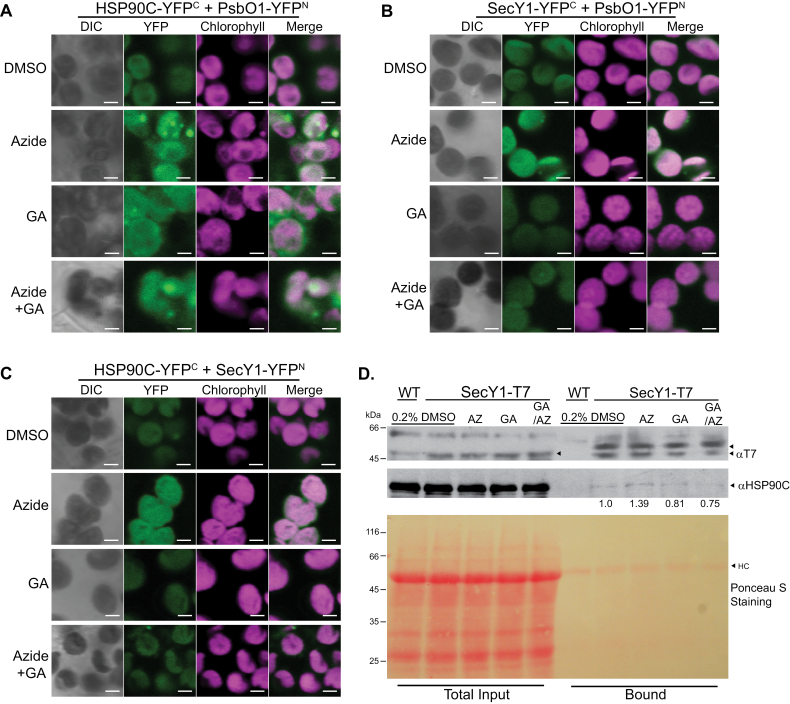
Effect of HSP90C and SEC translocon inhibitors on HSP90C–SecY1 interactions. Laser scanning microscopy images were taken from mesophyll cells of *N. benthamiana* transiently co-expressing YFP N- or C-half fusion proteins that are labeled as -YFP^N^ and -YFP^C^, respectively. Fluorescence images for YFP, chlorophyll, and the merged signals were shown. Scale bars represent 2.5 µm. Mesophyll chloroplasts were incubated with 30 mM geldanamycin (GA) and 10 mM sodium azide (Azide) separately. Phase contrast (DIC) and fluorescence images for YFP, chlorophyll, and the merged signals are shown. (A) Fluorescence signals from chloroplasts expressing HSP90C–YFP^C^ and PsbO1–YFP^N^ fusion proteins. (B) Fluorescence signals from chloroplasts expressing SecY1–YFP^C^ and PsbO1–YFP^N^ fusion proteins. (C) Fluorescence signals from chloroplasts expressing HSP90C–YFP^C^ and SecY1–YFP^N^ fusion proteins. (D) Co-IP of the SecY1 complex from 12-day-old *SECY1-T7*/*secy1* Arabidopsis seedlings after treatment with sodium azide (AZ), geldanamycin (GA), or both sodium azide and geldanamycin (AZ/GA) using anti-T7 antibody agarose. Wild-type (WT) seedlings were used as a negative control and 0.2% DMSO only as mock chemical treatment. Anti-HSP90C and anti-T7 antibodies were used to detect HSP90C and SecY1 proteins in both total input and the pull-down (IP) samples. The relative amounts of co-purified HSP90C normalized to the mock chemical treatment are indicated under the anti-HSP90C blot.

To determine how HSP90C activity affects interaction with SecY1, we analyzed the BiFC YFP fluorescence signals in the presence of sodium azide or geldanamycin. Both chemicals enhanced YFP signal when HSP90C and PsbO1 were paired (Fig. 7A), but the YFP signal was less localized to the thylakoid in the presence of azide. This result indicated that HSP90C did not require its active ATPase activity to interact with PsbO1. Azide also enhanced HSP90C–SecY1 interaction and PsbO1–SecY1 interaction, whereas geldanamycin reduced interactions between HSP90C and SecY1, and between PsbO1 and SecY1 (Fig. 7B, C). This suggests that the HSP90C ATPase activity is required for its efficient association with the SEC complex and that interaction might be mediated by a different factor. To further confirm the effect of azide and geldanamycin on HSP90C–SecY1 interaction *in vivo*, we decided to use SCY1–T7 Arabidopsis seedlings once again as previously shown (Fig. 6D), due to the ease of obtaining a large amount of samples. Although the anti-T7 antibody recognized multiple non-specific protein bands, it is clear that more HSP90C proteins were enriched with T7-tagged SecY1 after treatment with azide, while geldanamycin in the absence or presence of azide inhibited HSP90C–SECY1 interaction (Fig. 7D), thus supporting the observed BiFC fluorescence patterns (Fig. 7C).

## Discussion

**Fig. 8.**
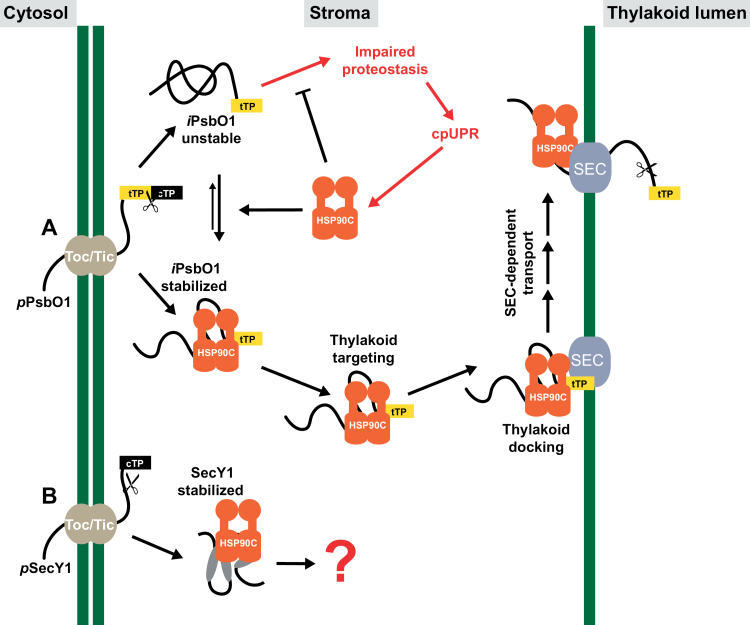
Model of HSP90C’s role in thylakoid SEC translocation. Under favorable photosynthetic conditions or during chloroplast maturation, *p*PsbO1 and *p*SecY1 are imported into the chloroplast stroma through the TOC/TIC translocon at the chloroplast envelope. (A) After import, *i*PsbO1 is stabilized by HSP90C and kept in a soluble, ready-to-transport state. Lack of HSP90C results in unstable *i*PsbO1 that can misfold, thus inducing the chloroplast UPR. HSP90C targets *i*PsbO1 towards the thylakoid and docks onto the SEC translocon. HSP90C probably keeps *i*PsbO1 in a conformation suitable for translocation into the thylakoid lumen. (B) SecY1 is stabilized by HSP90C and kept in a soluble, ready-to-transport state.

Protein homeostasis within the chloroplast stroma is primarily regulated by protein influx after cytoplasmic pre-protein synthesis, targeting, and import, and protein efflux or turnover that is mediated by subsequent trafficking to other subplastic compartments, such as the thylakoid, or by direct protein degradation. Incorrect protein homeostasis leads to accumulation of unfolded/misfolded/damaged proteins in the stroma that subsequently induce the chloroplast unfolded protein response (cpUPR), particularly when the protein degradation machinery such as FtsH2 or ClpP is defective (Llamas *et al.*, 2017; Dogra *et al.*, 2019). In this study, we investigated the thylakoid SEC translocation pathway and attempted to understand how stromal protein homeostasis is involved in the SEC-dependent protein trafficking process, with a focus on the role of the stromal molecular chaperone HSP90C. Studying the role of stromal factors in thylakoid protein trafficking *in vivo* is generally difficult given that drastically modifying thylakoid protein transport often has a negative effect on chloroplast biogenesis and plant development. In this report, we adopted an *in organello* chase assay using isolated chloroplasts which had accumulated precursors of some SEC substrate proteins naturally. We showed that HSP90C activity was necessary for SEC translocation into the lumen (Figs 1, 2). We also demonstrated that enhanced interaction between HSP90C and PsbO1 better facilitated this process by using a PsbO1 T200A mutant (Fig. 3). Analyzing the transport of endogenous substrates of the SEC, SRP, and TAT pathways in multiple transgenic lines suggests that HSP90C function is primarily affecting the SEC pathway, but not the TAT or SRP pathway (Fig. 4). We also showed that both PsbO1 and HSP90C directly interacted with SecY1 (Fig. 6). Additionally, blocking SEC-dependent protein efflux out of the stroma or inhibiting HSP90C activity altered not only the localization pattern of PsbO1 (Fig. 5), but also the interaction of PsbO1 or HSP90C with the SecY1 in the thylakoid (Fig. 7). This study confirms that HSP90C plays a critical role in chloroplast stromal protein homeostasis, and also provides evidence that it directly binds and targets proteins for SEC-dependent thylakoid trafficking. A summary model for the role of HSP90C in chloroplast stroma is therefore proposed (Fig. 8).

HSP90’s role in protein folding has been extensively studied for the cytosolic isoforms in mammalian and yeast cells (Prodromou, 2016; Schopf *et al.*, 2017; Biebl and Buchner, 2019). The broad and important roles of HSP90 in plants have also been long recognized and reviewed ( Queitsch *et al.*, 2002; Hubert *et al.*, 2003; Ticha *et al*., 2020). Arabidopsis plastid HSP90C was initially referred to as CR88 since Arabidopsis with a point mutation in this protein was chlorate resistant (Lin and Cheng, 1997; Cao *et al.*, 2003). However, the major role of HSP90C was later documented to form a foldosome with HSP70 in green algae (Schroda and Muhlhaus, 2009) and to aid in general chloroplast protein import in Arabidopsis (Inoue *et al.*, 2013). Although the spectrum of potential HSP90C substrate proteins has been investigated in multiple studies (Heide *et al.*, 2009; Inoue *et al.*, 2013; Feng *et al.*, 2014; Jiang *et al.*, 2017), we provide evidence here for the first time that HSP90C interacts with and directly targets proteins to the thylakoid Sec translocon, therefore further demonstrating the multifaceted role of the HSP90 family molecular chaperones in higher organisms.

Previously, we observed that HSP90C interacted with both the mature segment of PsbO1 and its thylakoid-targeting sequence *in vivo*. Here, we showed that HSP90C can interact with SecY1 as well, using a transient expression system in tobacco and co-IP analysis directly from Arabidopsis seedlings employing SecY1–T7 lines (Fig. 6). Interestingly, purification of the T7 epitope-tagged protein using anti-T7 antibody affinity resin resulted in two distinct bands, with a low molecular weight protein band being the dominant form (Fig. 6D). Overexpressed SecY1–GFP has been shown to reside within the stroma and is localized to the thylakoid specifically after exposure to light (Singhal and Fernandez, 2017). The presence of two bands may represent distinct thylakoid and stromal forms. It should be noted that HSP90C and its activity may function in the thylakoid integration of the stromal pool of SecY1 (Fig. 8). Integration of thylakoid membrane proteins typically involves the SRP pathway; however, Arabidopsis mutants lacking cpSRP54 do not exhibit reduced accumulation of SecY1 or of the SEC substrate PC (Hristou *et al.*, 2019). SecE1 thylakoid integration was also found to depend on unknown targeting aspects intrinsic to its transmembrane domain and C-terminal tail (Anderson *et al.*, 2019). While the involvement of HSP90C in these processes requires further investigation, it is not surprising that HSP90C plays many roles within chloroplasts.

SEC substrates must be translocated through SecY in an unfolded state. We found that HSP90C activity is necessary to prevent misfolding and aggregation of PsbO1 within the stroma. As an intrinsically disordered protein, PsbO1 must adopt a molten globule structure within the neutral stroma environment (Popelkova and Yocum, 2011). The endoplasmic reticulum (ER) Sec61 and its subcomplex components Sec71–Sec72 have substrates recruited either post-translationally or co-translationally by HSPs (Tripathi *et al.*, 2017). Similar scenarios have been observed in bacteria where DnaK (Hsp70), DnaJ (Hsp40), GroEL/ES (Hsp60/Hsp10), and SecB are required for transport of Sec substrate proteins (Phillips and Silhavy, 1990; Sakr *et al.*, 2010; Calloni *et al.*, 2012; Castanie-Cornet *et al*., 2014). Given that both SecY and ER Sec61 are inherently deficient in translocating intrinsically disordered proteins (Gonsberg *et al.*, 2017; Jung and Tatzelt, 2018), HSP90C might serve to keep *i*PsbO1 in a ready-to-transport state within chloroplast stroma.

We previously showed that the SEC translocon-targeting transit peptide of PsbO1 (amino acids 59–86) interacted with the HSP90C–cpHSP70 complex (Jiang *et al.*, 2017). SEC signal peptides generally do not show sequence similarities; however, a conserved tripartite structure can be recognized, including a positively charged N-terminal region, a central hydrophobic core, and a polar C-terminal domain that contains the signal peptidase recognition site (consensus motif A-X-A) (Rusch and Kendall, 2007). While the positive N-terminal region and polar C-terminal domain are not as obvious in plants, the hydrophobic middle domain, which is known to promote membrane translocation (Chou and Kendall, 1990; Goldstein *et al.*, 1991), is obvious among the tTPs of PsbO1 and PC. Cytosolic HSP70 have been previously proposed to interact with the cTP to promote pre-protein guiding (Chotewutmontri and Bruce, 2015). The HSP90C–cpHSP70 complex probably functions in a similar manner to guide SEC substrates from the TIC to the SEC.

Finally, the classical technique to study protein trafficking across the thylakoid membrane is to monitor the transport of radiolabeled and *in vitro* translated pre-proteins over time. This technique disrupts the chloroplast envelope and requires reconstitution of a stroma-like environment by using isolated stromal proteins. Here, we established an *in organello* thylakoid transport assay based on immunoblotting analysis by monitoring the transport of those naturally accumulated stromal PsbO1^59–85^GFP fusion proteins (Fig. 1). This *in organello* transport assay allowed us to specifically evaluate thylakoid transport with no need to consider protein import efficiency across the envelopes with *in vitro* translated or purified proteins. Furthermore, GFP led by the thylakoid targeting sequence interacts specifically with HSP90C and does not have severe aggregation or clustering phenotypes under normal conditions. While we did not conduct any assays using the PsbO1^59–332^GFP-containing chloroplasts, future analysis could be performed with this organelle model as HSP90C had higher affinity for *i*PsbO1^1–332^GFP than for PsbO1^59–85^GFP (Jiang *et al.*, 2017). The same assay could be further combined with fluorescence imaging analysis after chemical treatment to understand the role of other chloroplast factors in protein stability and protein aggregation.

## Supplementary data

The following supplementary data are available at *JXB* online.

Table S1. Primers used to clone BiFC vectors into pDONR207.

Table S2. Plasmid constructs used and generated in this study.

Fig. S1. Fractionation of intact chloroplasts and treatment of thylakoids with proteases.

Fig. S2. Immunoblotting analysis of fractionated thylakoids after *in organello* chase and treatment with thermolysin.

Fig. S3. Sequence alignment of PsbO proteins.

Fig. S4. Accumulation of representative plastid proteins during photomorphogenesis.

Fig. S5. Expression of representative chaperone proteins during photomorphogenesis.

Fig. S6. Fractionation of isolated tobacco chloroplasts after transient expression of PsbO1^1–85^GFP fusion proteins.

Fig. S7. Co-localization of tobacco mesophyll chloroplast stromal-targeted CFP with PsbO1^1–332^YFP clusters.

Fig. S8. Immunoblotting analysis of infiltrated tobacco leaf total soluble proteins.

Fig. S9. Bifluorescence complementation assay between HSP90C and RbcS3B.

eraa399_suppl_Supplementary_File001

## Data Availability

All data supporting the findings of this study are available within the paper and within its supplementary data published online. All constructs and plants lines created in this study are available on request from the corresponding author RZ.

## Abbreviations

GFP: green fluorescent protein
HSP90C: plastid heat shock protein 90
*i*GFP: stromal intermediate GFP fusion
*i*PsbO1: stromal intermediate form of PsbO1
*m*GFP: thylakoid lumen located mature form of GFP
*m*PsbO1: mature form PsbO1
pPsbO1: pre-protein form of PsbO1
PsbO1: PSII subunit O isoform 1

## References

[CIT0001] Albiniak AM , Baglieri J, Robinson C. 2012. Targeting of lumenal proteins across the thylakoid membrane. Journal of Experimental Botany 63, 1689–1698. 22275386 10.1093/jxb/err444

[CIT0002] Anderson SA , Singhal R, Fernandez DE. 2019. Membrane-specific targeting of tail-anchored proteins SECE1 and SECE2 within chloroplasts. Frontiers in Plant Science 10, 1401. 31781139 10.3389/fpls.2019.01401 PMC6857650

[CIT0003] Armarego-Marriott T , Kowalewska Ł, Burgos A, et al. 2019. Highly resolved systems biology to dissect the etioplast-to-chloroplast transition in tobacco leaves. Plant Physiology 180, 654–681. 30862726 10.1104/pp.18.01432 PMC6501100

[CIT0004] Bechtluft P , van Leeuwen RG, Tyreman M, Tomkiewicz D, Nouwen N, Tepper HL, Driessen AJ, Tans SJ. 2007. Direct observation of chaperone-induced changes in a protein folding pathway. Science 318, 1458–1461. 18048690 10.1126/science.1144972

[CIT0005] Biebl MM , Buchner J. 2019. Structure, function, and regulation of the Hsp90 machinery. Cold Spring Harbor Perspectives in Biology 11, a034017. 30745292 10.1101/cshperspect.a034017 PMC6719599

[CIT0006] Calloni G , Chen T, Schermann SM, Chang HC, Genevaux P, Agostini F, Tartaglia GG, Hayer-Hartl M, Hartl FU. 2012. DnaK functions as a central hub in the *E. coli* chaperone network. Cell Reports 1, 251–264. 22832197 10.1016/j.celrep.2011.12.007

[CIT0007] Cao D , Froehlich JE, Zhang H, Cheng CL. 2003. The chlorate-resistant and photomorphogenesis-defective mutant cr88 encodes a chloroplast-targeted HSP90. The Plant Journal 33, 107–118. 12943545 10.1046/j.1365-313x.2003.016011.x

[CIT0008] Cao D , Lin Y, Cheng CL. 2000. Genetic interactions between the chlorate-resistant mutant cr 8 8 and the photomorphogenic mutants cop1 and hy5. The Plant Cell 12, 199–210. 10662857 10.1105/tpc.12.2.199 PMC139758

[CIT0009] Castanié-Cornet MP , Bruel N, Genevaux P. 2014. Chaperone networking facilitates protein targeting to the bacterial cytoplasmic membrane. Biochimica et Biophysica Acta 1843, 1442–1456. 24269840 10.1016/j.bbamcr.2013.11.007

[CIT0010] Chen YE , Yuan S, Schröder WP. 2016. Comparison of methods for extracting thylakoid membranes of Arabidopsis plants. Physiologia Plantarum 156, 3–12. 26337850 10.1111/ppl.12384

[CIT0011] Chen YL , Chen LJ, Li HM. 2016. Polypeptide transport-associated domains of the Toc75 channel protein are located in the intermembrane space of chloroplasts. Plant Physiology 172, 235–243. 27388682 10.1104/pp.16.00919 PMC5074630

[CIT0012] Chotewutmontri P , Bruce BD. 2015. Non-native, N-terminal Hsp70 molecular motor recognition elements in transit peptides support plastid protein translocation. Journal of Biological Chemistry 290, 7602–7621. 10.1074/jbc.M114.633586 PMC4367265 25645915

[CIT0013] Chou MM , Kendall DA. 1990. Polymeric sequences reveal a functional interrelationship between hydrophobicity and length of signal peptides. Journal of Biological Chemistry 265, 2873–2880. 2154463

[CIT0014] Dogra V , Duan J, Lee KP, Kim C. 2019. Impaired PSII proteostasis triggers a UPR-like response in the var2 mutant of Arabidopsis. Journal of Experimental Botany 70, 3075–3088. 30989223 10.1093/jxb/erz151 PMC6598079

[CIT0015] Duong S , Vonapartis E, Li CY, Patel S, Gazzarrini S. 2017. The E3 ligase ABI3-INTERACTING PROTEIN2 negatively regulates FUSCA3 and plays a role in cotyledon development in *Arabidopsis thaliana*. Journal of Experimental Botany 68, 1555–1567. 28369580 10.1093/jxb/erx046 PMC5441903

[CIT0016] Endow JK , Singhal R, Fernandez DE, Inoue K. 2015. Chaperone-assisted post-translational transport of plastidic type i signal peptidase 1. Journal of Biological Chemistry 290, 28778–28791. 10.1074/jbc.M115.684829 PMC4661394 26446787

[CIT0017] Feng J , Fan P, Jiang P, Lv S, Chen X, Li Y. 2014. Chloroplast-targeted Hsp90 plays essential roles in plastid development and embryogenesis in Arabidopsis possibly linking with VIPP1. Physiologia Plantarum 150, 292–307. 23875936 10.1111/ppl.12083

[CIT0018] Flores-Pérez Ú , Bédard J, Tanabe N, Lymperopoulos P, Clarke AK, Jarvis P. 2016. Functional analysis of the Hsp93/ClpC chaperone at the chloroplast envelope. Plant Physiology 170, 147–162. 26586836 10.1104/pp.15.01538 PMC4704595

[CIT0019] Frain KM , Gangl D, Jones A, Zedler JA, Robinson C. 2016. Protein translocation and thylakoid biogenesis in cyanobacteria. Biochimica et Biophysica Acta 1857, 266–273. 26341016 10.1016/j.bbabio.2015.08.010

[CIT0020] Ganesan I , Shi LX, Labs M, Theg SM. 2018. Evaluating the functional pore size of chloroplast TOC and TIC protein translocons: import of folded proteins. The Plant Cell 30, 2161–2173. 30104404 10.1105/tpc.18.00427 PMC6181021

[CIT0021] Goldstein J , Lehnhardt S, Inouye M. 1991. In vivo effect of asparagine in the hydrophobic region of the signal sequence. Journal of Biological Chemistry 266, 14413–14417. 1860848

[CIT0022] Gonsberg A , Jung S, Ulbrich S, Origi A, Ziska A, Baier M, Koch HG, Zimmermann R, Winklhofer KF, Tatzelt J. 2017. The Sec61/SecY complex is inherently deficient in translocating intrinsically disordered proteins. Journal of Biological Chemistry 292, 21383–21396. 10.1074/jbc.M117.788067 PMC5766967 29084847

[CIT0023] Heide H , Nordhues A, Drepper F, Nick S, Schulz-Raffelt M, Haehnel W, Schroda M. 2009. Application of quantitative immunoprecipitation combined with knockdown and cross-linking to *Chlamydomonas* reveals the presence of vesicle-inducing protein in plastids 1 in a common complex with chloroplast HSP90C. Proteomics 9, 3079–3089. 19526558 10.1002/pmic.200800872

[CIT0024] Hristou A , Gerlach I, Stolle DS, Neumann J, Bischoff A, Dünschede B, Nowaczyk MM, Zoschke R, Schünemann D. 2019. Ribosome-associated chloroplast SRP54 enables efficient cotranslational membrane insertion of key photosynthetic proteins. The Plant Cell 31, 2734–2750. 31444312 10.1105/tpc.19.00169 PMC6881123

[CIT0025] Huang PK , Chan PT, Su PH, Chen LJ, Li HM. 2016. Chloroplast Hsp93 directly binds to transit peptides at an early stage of the preprotein import process. Plant Physiology 170, 857–866. 26676256 10.1104/pp.15.01830 PMC4734592

[CIT0026] Hubert DA , Tornero P, Belkhadir Y, Krishna P, Takahashi A, Shirasu K, Dangl JL. 2003. Cytosolic HSP90 associates with and modulates the Arabidopsis RPM1 disease resistance protein. The EMBO Journal 22, 5679–5689. 14592967 10.1093/emboj/cdg547 PMC275404

[CIT0027] Inoue H , Li M, Schnell DJ. 2013. An essential role for chloroplast heat shock protein 90 (Hsp90C) in protein import into chloroplasts. Proceedings of the National Academy of Sciences, USA 110, 3173–3178. 10.1073/pnas.1219229110 PMC3581895 23382192

[CIT0028] Islam S , Bhor SA, Tanaka K, Sakamoto H, Yaeno T, Kaya H, Kobayashi K. 2020. Impaired expression of chloroplast HSP90C chaperone activates plant defense responses with a possible link to a disease-symptom-like phenotype. International Journal of Molecular Sciences 21, 4202. 10.3390/ijms21124202 PMC7352560 32545608

[CIT0029] Jarvis P , López-Juez E. 2013. Biogenesis and homeostasis of chloroplasts and other plastids. Nature Reviews. Molecular Cell Biology 14, 787–802. 24263360 10.1038/nrm3702

[CIT0030] Jiang T , Oh ES, Bonea D, Zhao R. 2017. HSP90C interacts with PsbO1 and facilitates its thylakoid distribution from chloroplast stroma in Arabidopsis. PLoS One 12, e0190168. 29281724 10.1371/journal.pone.0190168 PMC5745004

[CIT0031] Jung S , Tatzelt J. 2018. Impaired transport of intrinsically disordered proteins through the Sec61 and SecY translocon; implications for prion diseases. Prion 12, 88–92. 29388511 10.1080/19336896.2018.1435936 PMC6016518

[CIT0032] Kikuchi S , Asakura Y, Imai M, et al. 2018. A Ycf2–FtsHi heteromeric AAA-ATPase complex is required for chloroplast protein import. The Plant Cell 30, 2677–2703. 30309901 10.1105/tpc.18.00357 PMC6305978

[CIT0033] Lee DW , Yoo YJ, Razzak MA, Hwang I. 2018. Prolines in transit peptides are crucial for efficient preprotein translocation into chloroplasts. Plant Physiology 176, 663–677. 29158328 10.1104/pp.17.01553 PMC5761803

[CIT0034] Lewis JD , Abada W, Ma W, Guttman DS, Desveaux D. 2008. The HopZ family of *Pseudomonas syringae* type III effectors require myristoylation for virulence and avirulence functions in *Arabidopsis thaliana*. Journal of Bacteriology 190, 2880–2891. 18263728 10.1128/JB.01702-07 PMC2293245

[CIT0035] Lin Y , Cheng CL. 1997. A chlorate-resistant mutant defective in the regulation of nitrate reductase gene expression in Arabidopsis defines a new HY locus. The Plant Cell 9, 21–35. 9014362 10.1105/tpc.9.1.21 PMC156898

[CIT0036] Llamas E , Pulido P, Rodriguez-Concepcion M. 2017. Interference with plastome gene expression and Clp protease activity in Arabidopsis triggers a chloroplast unfolded protein response to restore protein homeostasis. PLoS Genetics 13, e1007022. 28937985 10.1371/journal.pgen.1007022 PMC5627961

[CIT0037] May T , Soll J. 2000. 14-3-3 proteins form a guidance complex with chloroplast precursor proteins in plants. The Plant Cell 12, 53–64. 10634907 10.1105/tpc.12.1.53 PMC140214

[CIT0038] Moore M , Harrison MS, Peterson EC, Henry R. 2000. Chloroplast Oxa1p homolog albino3 is required for post-translational integration of the light harvesting chlorophyll-binding protein into thylakoid membranes. Journal of Biological Chemistry 275, 1529–1532. 10.1074/jbc.275.3.1529 10636840

[CIT0039] Mori H , Summer EJ, Ma X, Cline K. 1999. Component specificity for the thylakoidal Sec and Delta pH-dependent protein transport pathways. Journal of Cell Biology 146, 45–56. 10.1083/jcb.146.1.45 PMC2199744 10402459

[CIT0040] Oh SE , Yeung C, Babaei-Rad R, Zhao R. 2014. Cosuppression of the chloroplast localized molecular chaperone HSP90.5 impairs plant development and chloroplast biogenesis in Arabidopsis. BMC Research Notes 7, 643. 25216779 10.1186/1756-0500-7-643 PMC4168064

[CIT0041] O’Neil PK , Richardson LGL, Paila YD, Piszczek G, Chakravarthy S, Noinaj N, Schnell D. 2017. The POTRA domains of Toc75 exhibit chaperone-like function to facilitate import into chloroplasts. Proceedings of the National Academy of Sciences, USA 114, E4868–E4876. 10.1073/pnas.1621179114 PMC5474763 28559331

[CIT0042] Petsalaki EI , Bagos PG, Litou ZI, Hamodrakas SJ. 2006. PredSL: a tool for the N-terminal sequence-based prediction of protein subcellular localization. Genomics, Proteomics & Bioinformatics 4, 48–55. 10.1016/S1672-0229(06)60016-8 PMC5054032 16689702

[CIT0043] Phillips GJ , Silhavy TJ. 1990. Heat-shock proteins DnaK and GroEL facilitate export of LacZ hybrid proteins in *E. coli*. Nature 344, 882–884. 2109835 10.1038/344882a0

[CIT0044] Popelkova H , Yocum CF. 2011. PsbO, the manganese-stabilizing protein: analysis of the structure–function relations that provide insights into its role in photosystem II. Journal of Photochemistry and Photobiology. B, Biology 104, 179–190. 10.1016/j.jphotobiol.2011.01.015 21316983

[CIT0045] Prodromou C . 2016. Mechanisms of Hsp90 regulation. The Biochemical Journal 473, 2439–2452. 27515256 10.1042/BCJ20160005 PMC4980810

[CIT0046] Qbadou S , Becker T, Mirus O, Tews I, Soll J, Schleiff E. 2006. The molecular chaperone Hsp90 delivers precursor proteins to the chloroplast import receptor Toc64. The EMBO Journal 25, 1836–1847. 16619024 10.1038/sj.emboj.7601091 PMC1456943

[CIT0047] Queitsch C , Sangster TA, Lindquist S. 2002. Hsp90 as a capacitor of phenotypic variation. Nature 417, 618–624. 12050657 10.1038/nature749

[CIT0048] Richter S , Lamppa GK. 1998. A chloroplast processing enzyme functions as the general stromal processing peptidase. Proceedings of the National Academy of Sciences, USA 95, 7463–7468. 10.1073/pnas.95.13.7463 PMC22651 9636172

[CIT0049] Roe SM , Prodromou C, O’Brien R, Ladbury JE, Piper PW, Pearl LH. 1999. Structural basis for inhibition of the Hsp90 molecular chaperone by the antitumor antibiotics radicicol and geldanamycin. Journal of Medicinal Chemistry 42, 260–266. 9925731 10.1021/jm980403y

[CIT0050] Rospert S , Glick BS, Jenö P, Schatz G, Todd MJ, Lorimer GH, Viitanen PV. 1993. Identification and functional analysis of chaperonin 10, the groES homolog from yeast mitochondria. Proceedings of the National Academy of Sciences, USA 90, 10967–10971. 10.1073/pnas.90.23.10967 PMC47902 7902576

[CIT0051] Rusch SL , Kendall DA. 2007. Interactions that drive Sec-dependent bacterial protein transport. Biochemistry 46, 9665–9673. 17676771 10.1021/bi7010064 PMC2675607

[CIT0052] Rütgers M , Muranaka LS, Schulz-Raffelt M, Thoms S, Schurig J, Willmund F, Schroda M. 2017. Not changes in membrane fluidity but proteotoxic stress triggers heat shock protein expression in *Chlamydomonas reinhardtii*. Plant, Cell & Environment 40, 2987–3001. 10.1111/pce.13060 28875560

[CIT0053] Sakr S , Cirinesi AM, Ullers RS, Schwager F, Georgopoulos C, Genevaux P. 2010. Lon protease quality control of presecretory proteins in *Escherichia coli* and its dependence on the SecB and DnaJ (Hsp40) chaperones. Journal of Biological Chemistry 285, 23506–23514. 10.1074/jbc.M110.133058 PMC2906341 20504766

[CIT0054] Schopf FH , Biebl MM, Buchner J. 2017. The HSP90 chaperone machinery. Nature Reviews. Molecular Cell Biology 18, 345–360. 28429788 10.1038/nrm.2017.20

[CIT0055] Schroda M , Mühlhaus T. 2009. A ‘foldosome’ in the chloroplast? Plant Signaling & Behavior 4, 301–303. 19794845 10.4161/psb.4.4.7758 PMC2664489

[CIT0056] Schünemann D . 2007. Mechanisms of protein import into thylakoids of chloroplasts. Biological Chemistry 388, 907–915. 17696774 10.1515/BC.2007.111

[CIT0057] Schuenemann D , Amin P, Hartmann E, Hoffman NE. 1999. Chloroplast SecY is complexed to SecE and involved in the translocation of the 33-kDa but not the 23-kDa subunit of the oxygen-evolving complex. Journal of Biological Chemistry 274, 12177–12182. 10.1074/jbc.274.17.12177 10207046

[CIT0058] Sela A , Piskurewicz U, Megies C, Mène-Saffrané L, Finazzi G, Lopez-Molina L. 2020. Embryonic photosynthesis affects post-germination plant growth. Plant Physiology 182, 2166–2181. 32060052 10.1104/pp.20.00043 PMC7140907

[CIT0059] Settles AM , Yonetani A, Baron A, Bush DR, Cline K, Martienssen R. 1997. Sec-independent protein translocation by the maize Hcf106 protein. Science 278, 1467–1470. 9367960 10.1126/science.278.5342.1467

[CIT0060] Shi LX , Theg SM. 2010. A stromal heat shock protein 70 system functions in protein import into chloroplasts in the moss *Physcomitrella patens*. The Plant Cell 22, 205–220. 20061551 10.1105/tpc.109.071464 PMC2828695

[CIT0061] Singhal R , Fernandez DE. 2017. Sorting of SEC translocase SCY components to different membranes in chloroplasts. Journal of Experimental Botany 68, 5029–5043. 28992187 10.1093/jxb/erx318 PMC5853536

[CIT0062] Skalitzky CA , Martin JR, Harwood JH, Beirne JJ, Adamczyk BJ, Heck GR, Cline K, Fernandez DE. 2011. Plastids contain a second sec translocase system with essential functions. Plant Physiology 155, 354–369. 21051552 10.1104/pp.110.166546 PMC3075773

[CIT0063] Su PH , Li HM. 2010. Stromal Hsp70 is important for protein translocation into pea and Arabidopsis chloroplasts. The Plant Cell 22, 1516–1531. 20484004 10.1105/tpc.109.071415 PMC2899880

[CIT0064] Tichá T , Samakovli D, Kuchařová A, Vavrdová T, Šamaj J. 2020. Multifaceted roles of HEAT SHOCK PROTEIN 90 molecular chaperones in plant development. Journal of Experimental Botany 71, 3966–3985. 32293686 10.1093/jxb/eraa177

[CIT0065] Tripathi A , Mandon EC, Gilmore R, Rapoport TA. 2017. Two alternative binding mechanisms connect the protein translocation Sec71–Sec72 complex with heat shock proteins. Journal of Biological Chemistry 292, 8007–8018. 10.1074/jbc.M116.761122 PMC5427277 28286332

[CIT0066] Trösch R , Töpel M, Flores-Pérez Ú, Jarvis P. 2015. Genetic and physical interaction studies reveal functional similarities between ALBINO3 and ALBINO4 in Arabidopsis. Plant Physiology 169, 1292–1306. 26265777 10.1104/pp.15.00376 PMC4587442

[CIT0067] Tsirigotaki A , De Geyter J, Šoštaric N, Economou A, Karamanou S. 2017. Protein export through the bacterial Sec pathway. Nature Reviews. Microbiology 15, 21–36. 27890920 10.1038/nrmicro.2016.161

[CIT0068] Tsuda K , Abraham-Juarez MJ, Maeno A, Dong Z, Aromdee D, Meeley R, Shiroishi T, Nonomura KI, Hake S. 2017. KNOTTED1 cofactors, BLH12 and BLH14, regulate internode patterning and vein anastomosis in maize. The Plant Cell 29, 1105–1118. 28381444 10.1105/tpc.16.00967 PMC5466031

[CIT0069] Walter B , Hristou A, Nowaczyk MM, Schunemann D. 2015. In vitro reconstitution of co-translational D1 insertion reveals a role of the cpSec-Alb3 translocase and Vipp1 in photosystem II biogenesis. The Biochemical Journal 468, 315–324. 25803492 10.1042/BJ20141425

[CIT0070] Yuan J , Cline K. 1994. Plastocyanin and the 33-kDa subunit of the oxygen-evolving complex are transported into thylakoids with similar requirements as predicted from pathway specificity. Journal of Biological Chemistry 269, 18463–18467. 8034593

[CIT0071] Yuan J , Henry R, McCaffery M, Cline K. 1994. SecA homolog in protein transport within chloroplasts: evidence for endosymbiont-derived sorting. Science 266, 796–798. 7973633 10.1126/science.7973633

[CIT0072] Zhang N , Xu J, Liu X, et al. 2019. Identification of HSP90C as a substrate of E3 ligase TaSAP5 through ubiquitylome profiling. Plant Science 287, 110170. 31481192 10.1016/j.plantsci.2019.110170

