# Supplementary material for: Plastid chaperone HSP90C guides precursor proteins to the SEC translocase for thylakoid transport
Source: J Exp Bot. 2020 Aug 27;71(22):7073–87. doi: 10.1093/jxb/eraa399 (PMC7906790; doi:10.1093/jxb/eraa399)
Supplement: eraa399_suppl_Supplementary_File001 [file eraa399_suppl_supplementary_file001.pdf]

Table S1. Primers used to clone BiFC vectors into pDONR207

Accession #	Gene	F/R	Primer sequence
AT5G66570	PsbO1	Forward	GGGG ACA AGT TTG TAC AAA AAA GCA GGC T ATGGCAGCCT CTCTCCAATC CA
AT5G66570	PsbO1^{59}	Reverse	GGGG AC CAC TTT GTA CAA GAA AGC TGG GTC GAAGTCCTTAAAGTCAGACTGGAA
AT5G66570	PsbO1^{332}	Reverse	GGGG AC CAC TTT GTA CAA GAA AGC TGG GTC CTCAAGTTGACCATACCACAC
AT2G04030	HSP90C	Forward	GGGG ACA AGT TTG TAC AAA AAA GCA GGC T ATGGCTCCTG CTTTGAGTAG AA
AT2G04030	HSP90C	Reverse	GGGG AC CAC TTT GTA CAA GAA AGC TGG GTC ATCTTGCCAAGGATCACTCT
AT2G18710	SecY1	Forward	GGGG ACA AGT TTG TAC AAA AAA GCA GGC T ATGATAACGG TAAGCGAAGT TT
AT2G18710	SecY1	Reverse	GGGG AC CAC TTT GTA CAA GAA AGC TGG GTC TGGATCATACTTGTCAAGCTCGT
AT5G38410	RbcS3B	Forward	GGGG ACA AGT TTG TAC AAA AAA GCA GGC T ATGGCTTCCTCTATGCT
AT5G38410	RbcS3B	Reverse	GGGG AC CAC TTT GTA CAA GAA AGC TGG GTC AGCTTCGGTGAAGC

Table S2. Plasmid constructs used and generated.

Accession #	Transgene	nYFP/cYFP	Reference
AT5G66570	pEGAD-PsbO1$^{1-59}$	Full-length GFP	Jiang et al, 2017
AT5G66570	pEGAD-PsbO1$^{1-86}$	Full-length GFP	Jiang et al, 2017
AT5G66570	pEGAD-PsbO1$^{1-332}$	Full-length GFP	Jiang et al, 2017
AT5G66570	pGWB540-PsbO1$^{1-332}$	Full-length YFP	This study
AT1G67090	pFGC -RbcS$^{1-79}$	Full-length CFP	Nelson et al, 2007
AT2G04030	pGWB402Ω -HSP90C	-	Oh et al, 2014
AT5G66570	pB7WGYn9-PsbO1$^{1-59}$	nYFP	This study
AT5G66570	pB7WGYc9-PsbO1$^{1-59}$	cYFP	This study
AT5G66570	pB7WGYn9-PsbO1$^{1-332}$	nYFP	This study
AT5G66570	pB7WGYc9-PsbO1$^{1-332}$	cYFP	This study
AT2G04030	pB7WGYn9-HSP90C	nYFP	This study
AT2G04030	pB7WGYc9-HSP90C	cYFP	This study
AT2G18710	pB7WGYn9-SecY1	nYFP	This study
AT2G18710	pB7WGYc9-SecY1	cYFP	This study
AT5G38410	pB7WGYc9-RbcS3B	nYFP	This study
AT5G38410	pB7WGYc9-RbcS3B	cYFP	This study

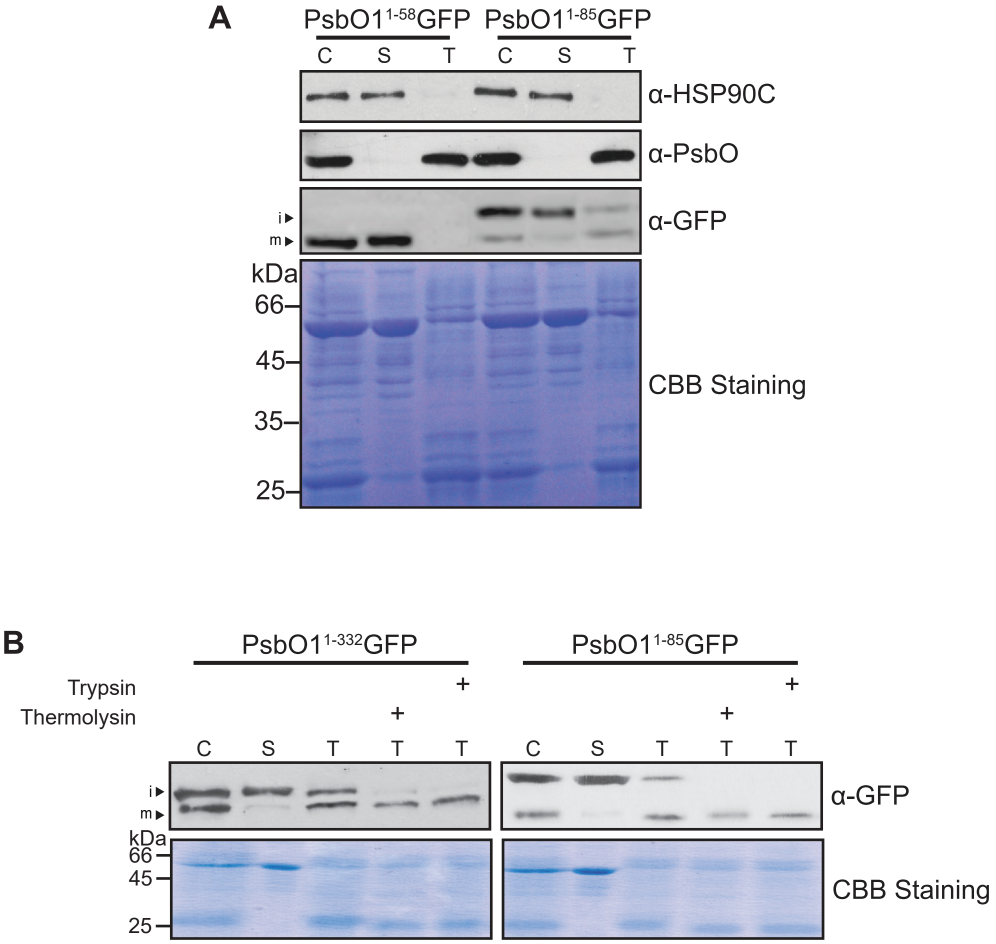

Figure S1. Fractionation of intact chloroplasts and treatment of thylakoid with proteases.
A. 12-DAG seedlings expressing PsbO1$^{1\text{-}58}$GFP and PsbO1$^{1\text{-}85}$GFP were grown under 16hr light / 8hr dark period. Intact chloroplasts (C) were isolated using a Percoll® gradient and fractionated into stroma (S) and thylakoid (T) using osmotic lysis. Samples were loaded equivalently.
B. Isolated thylakoids from PsbO1$^{1\text{-}332}$GFP and PsbO1$^{1\text{-}85}$GFP seedlings were treated with thermolysin and trypsin before loaded equivalently for immunoblotting with anti-GFP antibody. Coomassie brilliant blue (CBB) staining indicates equal loading.

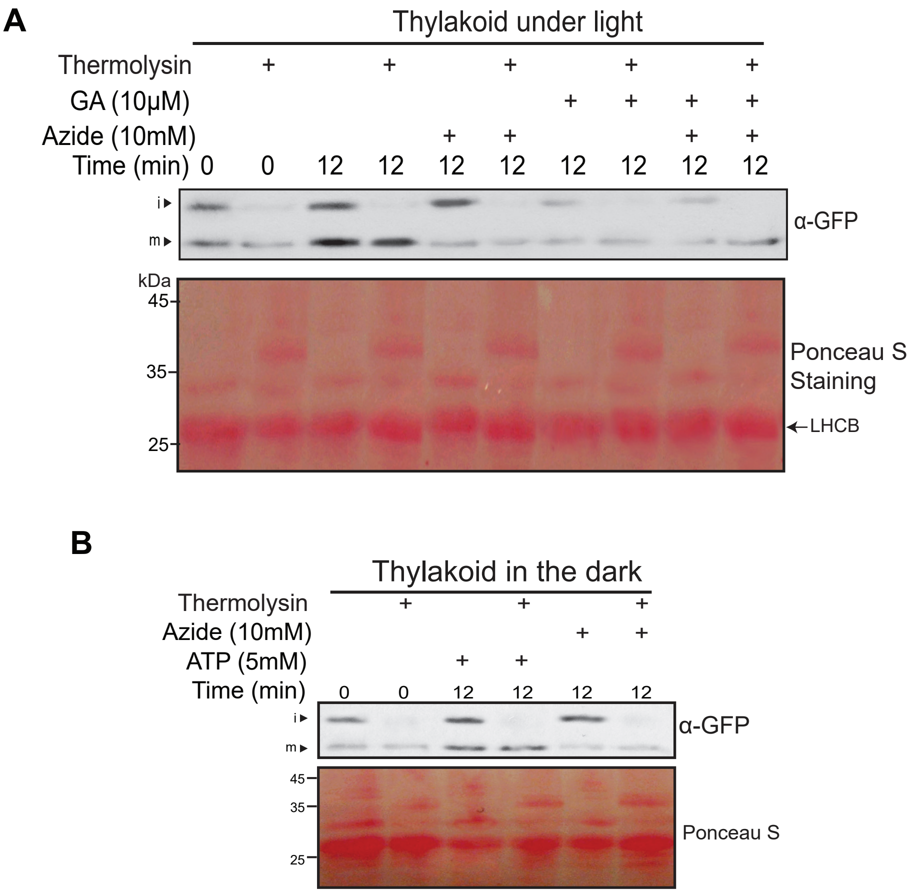

Figure S2. Immunoblotting analysis of fractionated thylakoid fraction after *in organello* chase assays and treatment with thermolysin.

A. Intact chloroplasts from PsbO1$^{1\text{-}85}$GFP expressing seedlings were used to chase the thylakoid transport of GFP protein under the light of 110 μmole.m^{-2}.s^{-1} at 22°C with or without the presence of geldanamycin or sodium azide, and then fractionated as shown in Figure 5A. Some thylakoid fractions were further treated with thermolysin (0.1mg/ml) before loading to SDS-PAGE and immunoblotted with anti-GFP antibody. Ponceau S staining images were used to indicated equivalent loading.

B. Intact chloroplasts from PsbO1$^{1\text{-}85}$GFP expressing seedlings were used to chase the thylakoid transport of GFP protein in the dark for 12 minutes with exogenous ATP and/or sodium azide included. After fractionation with osmolysis, some thylakoid samples from the chase assays were treated with thermolysin before loading to SDS-PAGE and immnoblotting with anti-GFP antibody.

```
                         10        20        30        40        50        60        70        80
                          |         |         |         |         |         |         |         |
PSBO_SOLTU       MAASLQAAATLMQPTKVGGVSARNNLQLRSSQSVSKAFGLEPSAS--RLSCSLQTDLKDFAQKCTDAAKIAGFALATSAL
PSBO_SOLLC       MAASLQAAATLMQPTKVG---VRNNLQLRSAQSVSKAFGVEQGSG--RLTCSLQTEIKELAQKCTDAAKIAGFALATSAL
I0B7J1_NICBE     MAVSLQAAATLMQPTKVGVAPARNNLQLRSAQSVCKAFGVEPAAA--RLTCSLQTELKDLAQKCTDAAKVAGFALATSAL
PSBO_SPIOL       MAASLQASTTFLQPTKVA---SRNTLQLRSTQNVCKAFGVESASSGGRLSLSLQSDLKELANKCVDATKLAGLALATSAL
PSBO1_ARATH      MAASLQSTATFLQSAKIATAPSRGSSHLRSTQAVGKSFGLETSSA--RLTCSFQSDFKDFTGKCSDAVKIAGFALATSAL
PSBO_THEEB       ---------------------------------------------------------------------MKYR-ILMATLLA
B8C4I5_THAPS     ---------------------------MKLVAALSLVAGVSAFAP-----AQTNSRSTQLNAMKDDIAKVGAAALASAAI
                                                                                     *      :*:

                         90       100       110       120       130       140       150       160
                          |         |         |         |         |         |         |         |
PSBO_SOLTU       VVSGANAEGVPKRLTFDEIQSKTYMEVKGTGTANQCPTIDGGV-DSFAFKPGK-YNAKKFCLEPTSFTVKAEG-VSKNSA
PSBO_SOLLC       VVSGANAEGVPKRLTYDEIQSKTYMEVKGTGTANQCPTIEGGV-GSFAFKPGK-YTAKKFCLEPTSFTVKAEG-VSKNSA
I0B7J1_NICBE     VVSGANAEGAPKRLTFDEIQSKTYMEVKGTGTANQCPTIEGGV-ASFAFKPGK-YNAKKFCLEPTSFTVKAES-VNKNAP
PSBO_SPIOL       IASGANAEGG-KRLTYDEIQSKTYLEVKGTGTANQCPTVEGGV-DSFAFKPGK-YTAKKFCLEPTKFAVKAEG-ISKNSG
PSBO1_ARATH      VVSGASAEGAPKRLTYDEIQSKTYMEVKGTGTANQCPTIDGGS-ETFSFKPGK-YAGKKFCFEPTSFTVKADS-VSKNAP
PSBO_THEEB       VCLGIFSLSA---PAFAAKQTLTYDDIVGTGLANKCPTLDDTARGAYPIDSSQTYRIARLCLQPTTFLVKEEP-KNKRQE
B8C4I5_THAPS     FGITAAPAQA---ITKSELNQLSYLQVKGTGLANRCSEVIGED--TITPKSGA--RLVDMCIEPKAWAVEEEIGKAGKTE
                 .     .       :    :  :* :: *** **:*. : .    : . ...       :*::*. : *: :     .

                        170       180       190       200       210       220       230       240
                          |         |         |         |         |         |         |         |
PSBO_SOLTU       PDFQKTKLMTRLTYTLDEIEGPFEVSPDGTVKFEEKDGIDYAAVTVQLPGGERVPFLFTIKQLVASGKP--------ESF
PSBO_SOLLC       PDFQKTKLMTRLTYTLDEIEGPFEVSPDGTVKFEEKDGIDYAAVTVQLPGGERVPFLFTIKQLVASGKP--------ESF
I0B7J1_NICBE     PDFQKTKLMTRLTYTLDEIEGPFEVSSDGTVKFEEKDGIDYAAVTVQLPGGERVPFLFTIKQLVASGKP--------ESF
PSBO_SPIOL       PDFQNTKLMTRLTYTLDEIEGPFEVSSDGTVKFEEKDGIDYAAVTVQLPGGERVPFLFTIKQLVASGKP--------ESF
PSBO1_ARATH      PEFQNTKLMTRLTYTLDEIEGPFEVASDGSVNFKEEDGIDYAAVTVQLPGGERVPFLFTVKQLDASGKP--------DSF
PSBO_THEEB       AEFVPTKLVTRETTSLDQIQGELKVNSDGSLTFVEEDGIDFQPVTVQMAGGERIPLLFTVKNLVASTQPNVTSITTSTDF
B8C4I5_THAPS     KKFVNSKVMTRQTYTLEGIEGSLKTDG-GKIVFTEEEGIDYAPTTVQLPGGERVPFLFTVKELVATGNGGAFKPG--FQM
                  .*  :*::** * :*: *:* ::.   *.: * *::***: ..***:.****:*:***:*:* *: :          .:

                        250       260       270       280       290       300       310       320
                          |         |         |         |         |         |         |         |
PSBO_SOLTU       SVDFLVPSYRGSSFLDPKGRGGSTGYDNAVALPAGGRGD--EEELQKENVKNTASLTGKITFTVTKSNPQTGEVIGVFES
PSBO_SOLLC       SGEFLVPSYRGSSFLDPKGRGGSTGYDNAVALPAGGRGD--EEELQKENVKNTASLTGKITLSVTQSKPETGEVIGVFES
I0B7J1_NICBE     SGEFLVPSYRGSSFLDPKGRGGSTGYDNAVALPAGGRGD--EEELEKENVKNTASSTGKITLSVTQSKPETGEVIGVFES
PSBO_SPIOL       SGDFLVPSYRGSSFLDPKGRGGSTGYDNAVALPAGGRGD--EEELQKENNKNVASSKGTITLSVTSSKPETGEVIGVFQS
PSBO1_ARATH      TGKFLVPSYRGSSFLDPKGRGGSTGYDNAVALPAGGRGD--EEELVKENVKNTAASVGEITLKVTKSKPETGEVIGVFES
PSBO_THEEB       KGEFNVPSYRTANFLDPKGRGLASGYDSAIALPQA-K----EEELARANVKRFSLTKGQISLNVAKVDGRTGEIAGTFES
B8C4I5_THAPS     GGDFSVPSYRTGLFLDPKGRGGVTGYDMAVALPGLQSGEEGDAELFKENNKTFEVGNGRIEMEVNKVNAEESEIGGVFVA
                   .* ***** . ********  :*** *:***        : ** : * *      * * : * . . . .*: *.* :

                        330       340
                          |         |
PSBO_SOLTU       IQPSDTDLGAKTPKDVKIQGIWYAQLES-
PSBO_SOLLC       IQPSDTDLGAKVPKDVKIQGIWYAQLE--
I0B7J1_NICBE     IQPSDTDLGAKVPKDVKIQGIWYAQLE--
PSBO_SPIOL       LQPSDTDLGAKVPKDVKIEGVWYAQLEQQ
PSBO1_ARATH      LQPSDTDLGAKVPKDVKIQGVWYGQLE--
PSBO_THEEB       EQLSDDDMGAHEPHEVKIQGVFYASIEPA
B8C4I5_THAPS     SQSGDTDMGSKVPKKILTKGIFYAKIQ--
                  * .* *:*:: *:.:  :*::*..::
```

Figure S3. Sequence alignment of PsbO proteins. PsbO sequences are from *Solanum tuberosum* (PsbO_SOLTU, P26320*)*, *Solanum lycopersicum* (PSBO_SOLLC, P23322), *Nicotiana benthamiana* (I0B7J1_NICBE, I0B7J1)*, Spinacia oleracea* (PSBO_SPIOL, P12359), Arabidopsis thaliana (PSBO1_ARATH, P23321), *Thermosynechococcus elongatus* (PSBO_THEEB, P0A431), and *Thalassiosira pseudonana* (B8C4I5_THAPS*,* B8C4I5). The second identifier in the bracket is the unique UniProtKB identifier). Thr200 in Arabidopsis thaliana PSBO1 was originally identified as an alanine (T200A) mutation from our original yeast two-hybrid screen and highlighted in yellow. "*" represents identical amino acids. "." and ":" represent similar amino acids. Alignment was performed using the CLUSTALW multiple alignment tool from https://npsa-prabi.ibcp.fr/cgi-bin/npsa_automat.pl?page=/NPSA/npsa_clustalw.html.

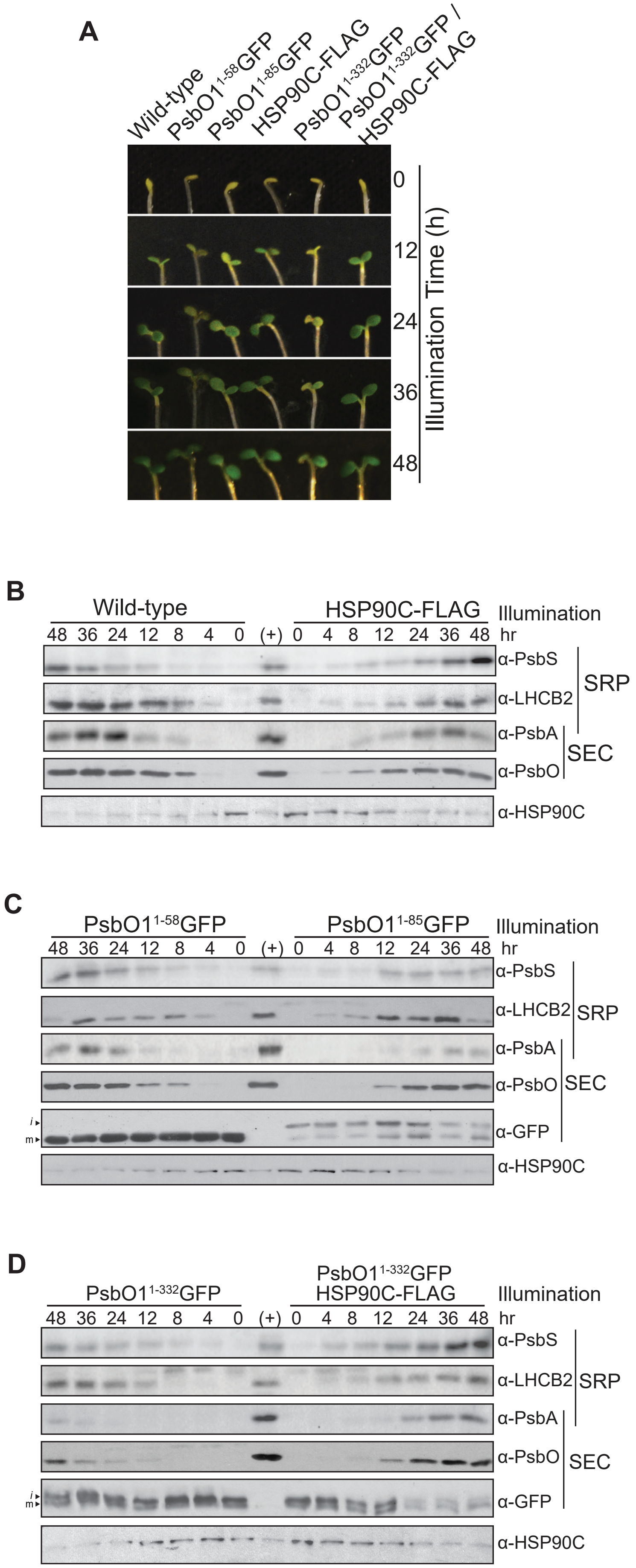
A
Wild-type
PsbO1^1-58GFP
PsbO1^1-85GFP
HSP90C-FLAG
PsbO1^1-332GFP
PsbO1^1-332GFP / HSP90C-FLAG
0
12
24
36
48
Illumination Time (h)
B
Wild-type
HSP90C-FLAG
Illumination
48 36 24 12 8 4 0 (+) 0 4 8 12 24 36 48 hr
α-PsbS
α-LHCB2
α-PsbA
α-PsbO
α-HSP90C
SRP
SEC
C
PsbO1^1-58GFP
PsbO1^1-85GFP
Illumination
48 36 24 12 8 4 0 (+) 0 4 8 12 24 36 48 hr
α-PsbS
α-LHCB2
α-PsbA
α-PsbO
α-GFP
α-HSP90C
SRP
SEC
i
m
D
PsbO1^1-332GFP
PsbO1^1-332GFP HSP90C-FLAG
Illumination
48 36 24 12 8 4 0 (+) 0 4 8 12 24 36 48 hr
α-PsbS
α-LHCB2
α-PsbA
α-PsbO
α-GFP
α-HSP90C
SRP
SEC
i
m

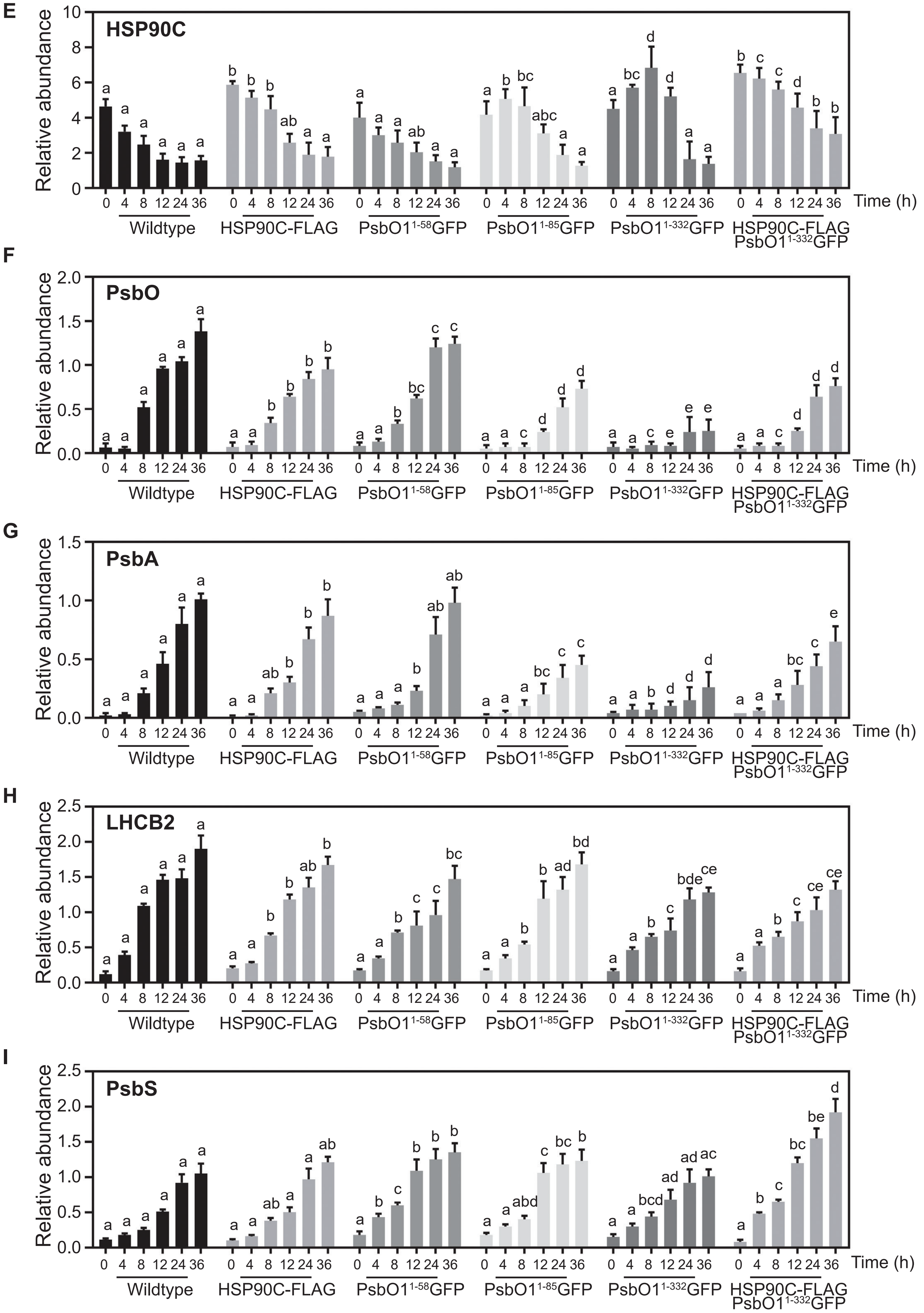
E
HSP90C
Relative abundance
0 2 4 6 8 10
a a a a a a
b b b ab a a
a a a ab a a
a b bc abc a a
a bc d d a a
b c c d b b
0 4 8 12 24 36 Time (h)
Wildtype
HSP90C-FLAG
PsbO1¹⁻⁵⁸GFP
PsbO1¹⁻⁸⁵GFP
PsbO1¹⁻³³²GFP
HSP90C-FLAG PsbO1¹⁻³³²GFP
F
PsbO
Relative abundance
0.0 0.5 1.0 1.5 2.0
a a a a a a
a a b b b b
a a b bc c c
a a c d d d
a a c e e e
a a c d d d
0 4 8 12 24 36 Time (h)
Wildtype
HSP90C-FLAG
PsbO1¹⁻⁵⁸GFP
PsbO1¹⁻⁸⁵GFP
PsbO1¹⁻³³²GFP
HSP90C-FLAG PsbO1¹⁻³³²GFP
G
PsbA
Relative abundance
0.0 0.5 1.0 1.5
a a a a a a
a a ab b b b
a a b ab ab
a a a bc c c
a a b d d d
a a a bc c e
0 4 8 12 24 36 Time (h)
Wildtype
HSP90C-FLAG
PsbO1¹⁻⁵⁸GFP
PsbO1¹⁻⁸⁵GFP
PsbO1¹⁻³³²GFP
HSP90C-FLAG PsbO1¹⁻³³²GFP
H
LHCB2
Relative abundance
0.0 0.5 1.0 1.5 2.0 2.5
a a a a a a
a a b b ab b
a a b c c bc
a a b b ad bd
a a b c bde ce
a a b c ce ce
0 4 8 12 24 36 Time (h)
Wildtype
HSP90C-FLAG
PsbO1¹⁻⁵⁸GFP
PsbO1¹⁻⁸⁵GFP
PsbO1¹⁻³³²GFP
HSP90C-FLAG PsbO1¹⁻³³²GFP
I
PsbS
Relative abundance
0.0 0.5 1.0 1.5 2.0 2.5
a a a a a a
a a ab a a ab
a b c b b b
a a abd c bc b
a a bcd ad ad ac
a b c bc be d
0 4 8 12 24 36 Time (h)
Wildtype
HSP90C-FLAG
PsbO1¹⁻⁵⁸GFP
PsbO1¹⁻⁸⁵GFP
PsbO1¹⁻³³²GFP
HSP90C-FLAG PsbO1¹⁻³³²GFP

Figure S4. Accumulation of plastid proteins during photomorphogenesis. A. Seeds were stratified and grown at 22°C in complete darkness for 3.5 days and then switched to constant light at 110 µmole.m^{-2}.s^{-1} for 48h. **B-D.** Immunoblots of photosynthetic proteins (LHCB2, PsbA, PsbS, PsbO), HSP90C and transgenic GFP fusion protein. Samples from wild-type, HSP90C-FLAG, PsbO1$^{1\text{-}58}$GFP, PsbO1$^{1\text{-}85}$GFP and PsbO1$^{1\text{-}332}$GFP co-expressing HSP90C-FLAG were taken and loaded based on equal total protein. Positive control for all samples come from 4-DAG wild-type seedlings grown under 16hr light / 8hr dark light cycle. Arrows labeled *i/m* represent intermediate/mature processing forms respectively. **E-I**. Quantitative analyses of HSP90C (**E**), PsbO (**F**), PsbA (**G**), LHCB2 (**H**) and PsbS (**I**) expression from the immunoblotting. Error bars represent standard deviation from 3 independent assays. Signals from the positive control loaded in the middle of the gel, indicated with (+) were used as references and set to 1.0. For each protein expressing in transgenic lines, their levels were compared to the one in wild type at the same time point and bars with different letters indicate they are significantly different. All time-point bars in wild type are labelled with "a" because they are used as references independently and not compared with one another.

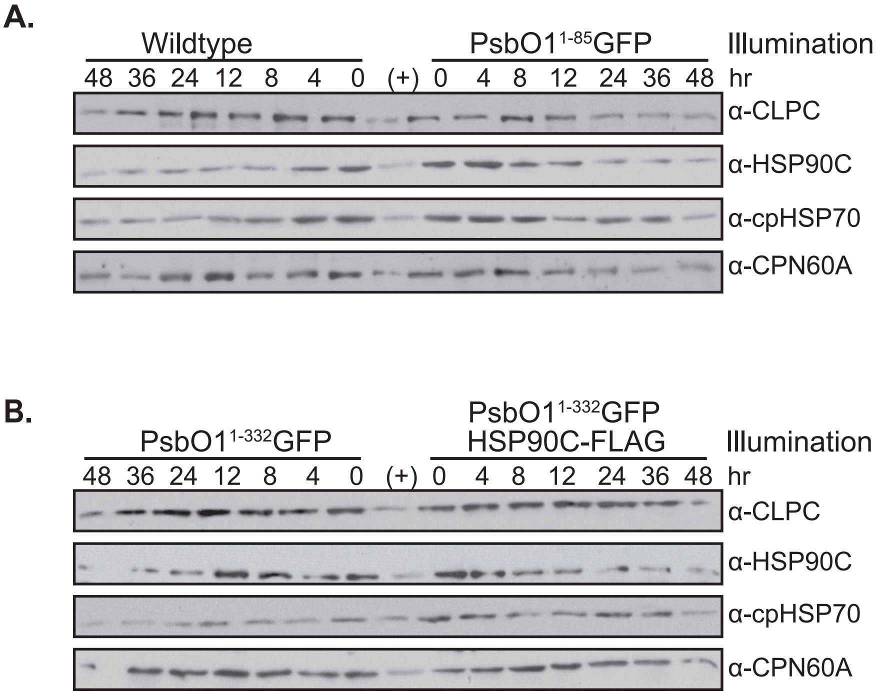

Figure S5. Accumulation of representative plastid chaperone proteins during photomorphogenesis.

Seeds were stratified and grown at 22°C in complete darkness for 3.5 days and then switched to constant light at 110 µmole.m^{-2}.s^{-1} for 48h. Protein samples were then made at different times and equivalent amounts were separated by SDS-PAGE and immunoblotted with anti-CLPC, anti-HSP90C, anti-cpHSP70 and anti-CPN60A antibodies. **A.** Samples from wild type seedlings and those expressing PsbO1$^{1\text{-}85}$GFP. **B.** Samples from seedlings expressing PsbO1$^{1\text{-}332}$GFP or PsbO1$^{1\text{-}332}$GFP /HSP90CFLAG. Positive control (+) for all samples comes from 4-DAG wild-type seedlings grown under 16hr light / 8hr dark light cycle.

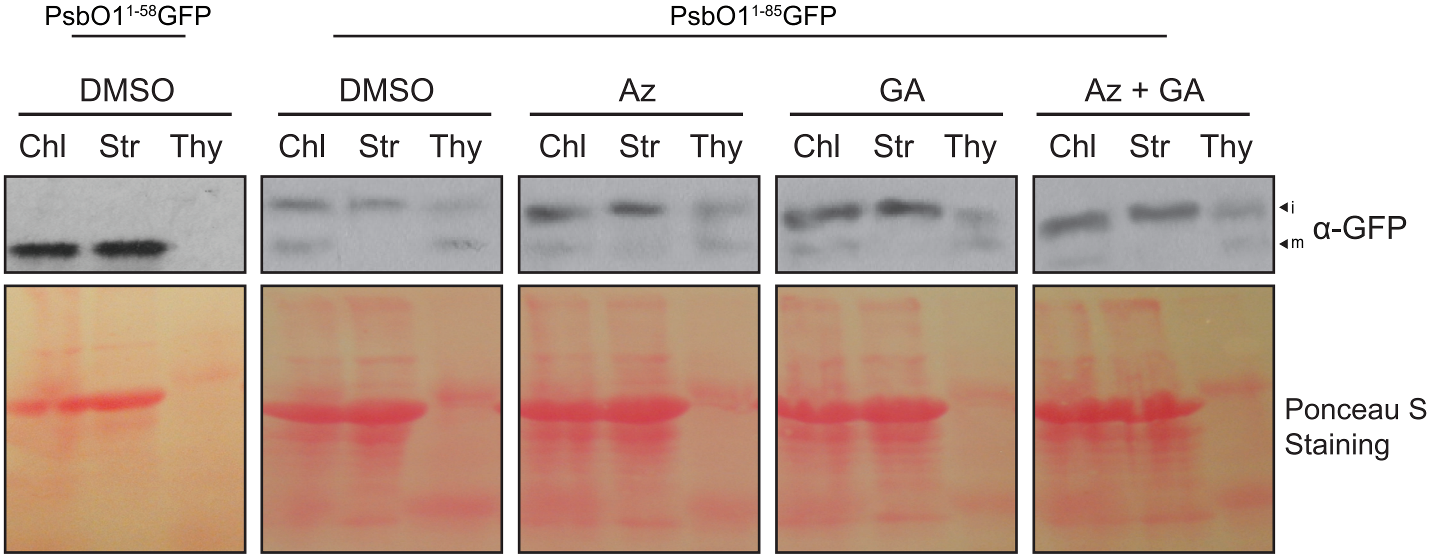

Figure S6. Fractionation of isolated tobacco chloroplasts after transient expression of PsbO1$^{1-85}$GFP fusion proteins.

Tobacco leaves transiently expressing PsbO1$^{1-58}$GFP and PsbO1$^{1-85}$GFP were collected and isolated for chloroplasts. The stroma and thylakoid fractions were then prepared and munobloted with anti-GFP. For some samples expressing PsbO1$^{1-85}$GFP, they were treated with 10 mM sodium azide (Az) or 30 μM geldanamycin (GA) or both together (Az +GA) before fractionation. Ponceau S staining images for blots were used to indicate equivalent loadings.

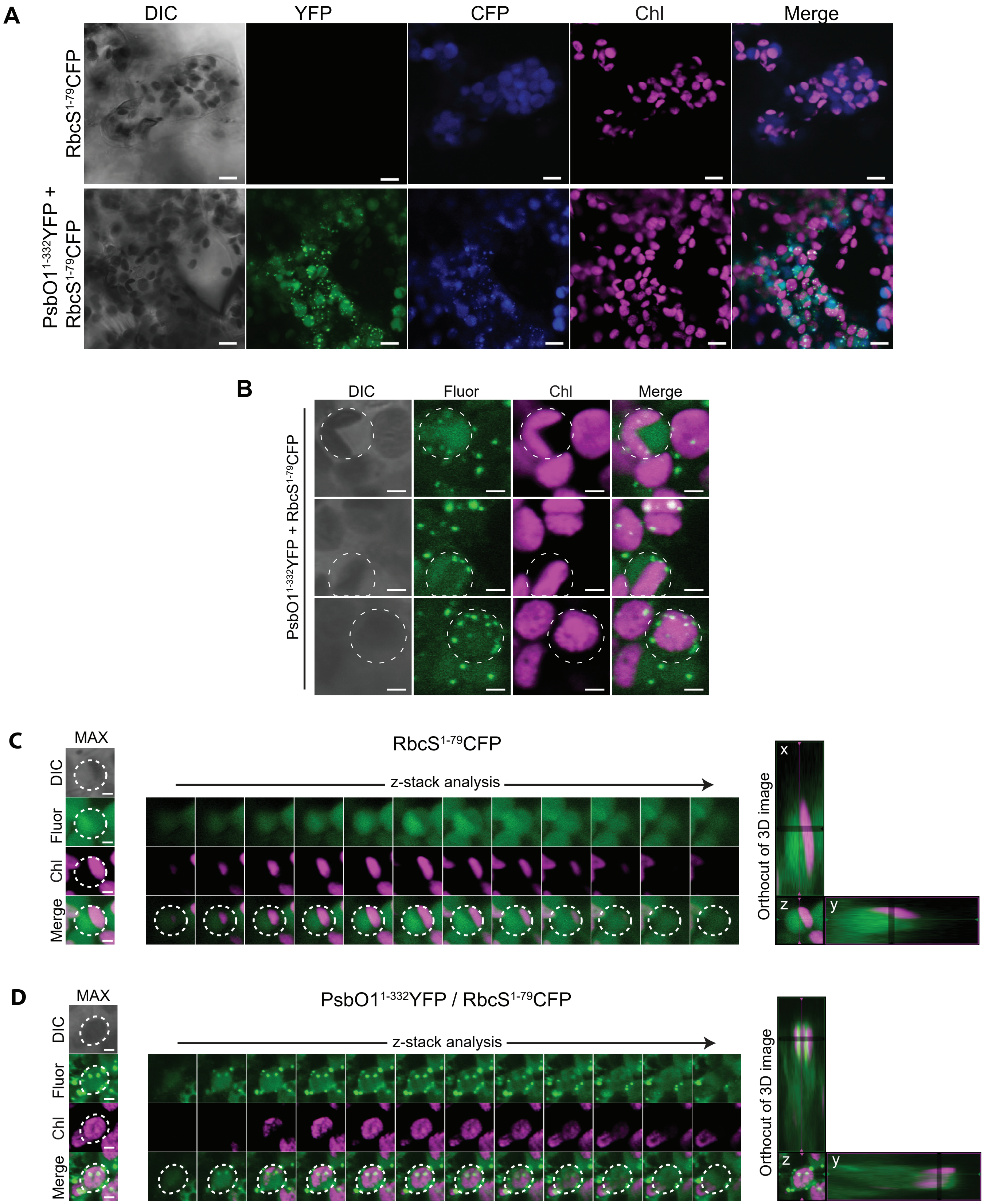

Figure. S7. Co-localization of tobacco mesophyll chloroplast stromal targeted CFP with PsbO1$^{1\text{-}332}$YFP clusters. A. Expression and co-expression of RbcS$^{1\text{-}79}$CFP and PsbO1$^{1\text{-}332}$YFP in mesophyll cells. Scale bar = 10µm. **B.** Zoomed images of chloroplasts having both stromal CFP and PsbO1$^{1\text{-}332}$YFP (Fluor represents CFP channel with leakage from YFP punctae). Dotted ovals represent individual chloroplast. Bars = 2.5µm. **C-D.** Z-stack analysis of chloroplasts containing RbcS$^{1\text{-}79}$CFP without/with PsbO1$^{1\text{-}332}$YFP. Dotted ovals represent individual chloroplast. Scale bar = 2.5µm.

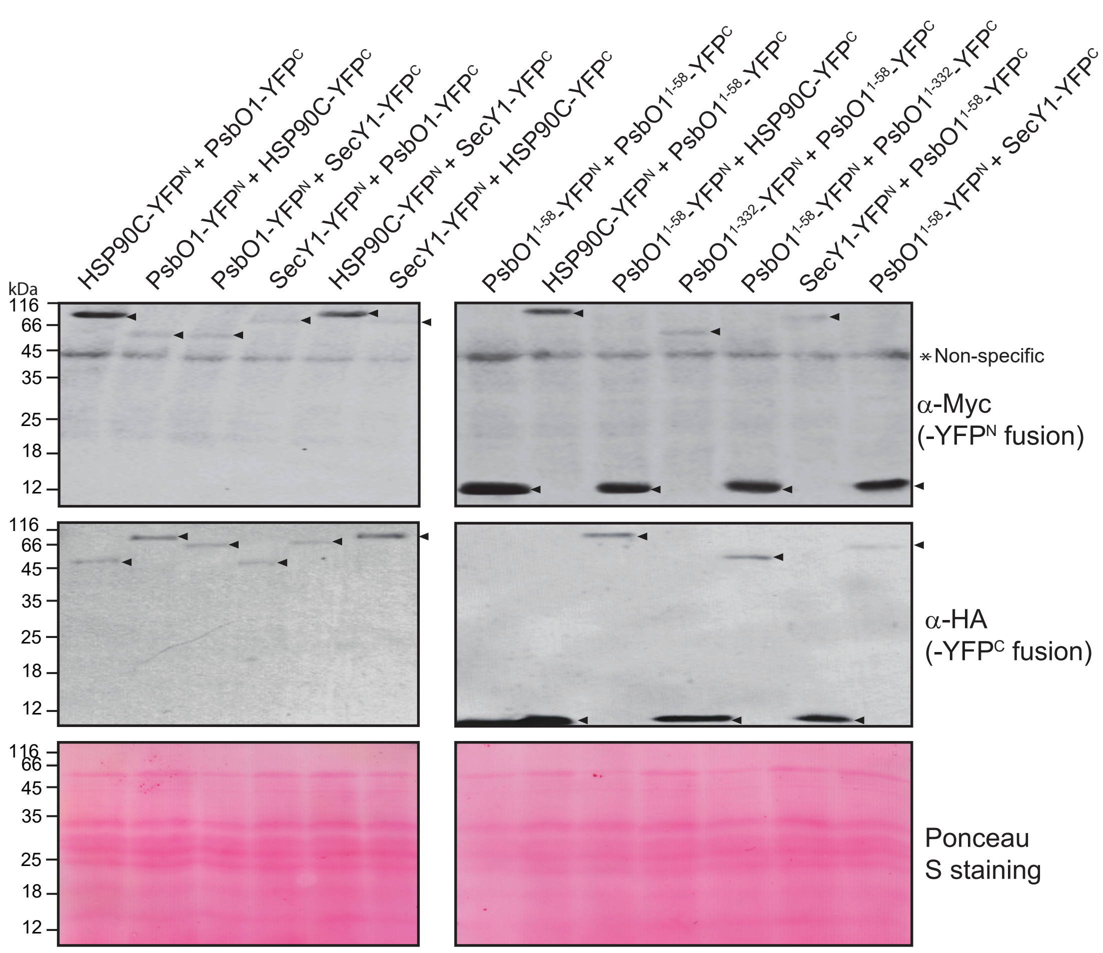

Figure S8. Immunoblotting the infiltrated tobacco leaf total soluble proteins. Tobacco leaves transiently expressing split YFP fusion proteins were collected and 15 μg total soluble proteins were loaded to SDS-PAGE and immunoblotted with anti-Myc for -YFP^{N} fusion proteins and anti-HA for YFP^{C} fusion proteins, as the two halves are also tagged with Myc and HA epitopes. Arrows indicate transiently expressed fusion protein bands according expected fusion protein size, and Ponceau S staining membranes show equal loading.

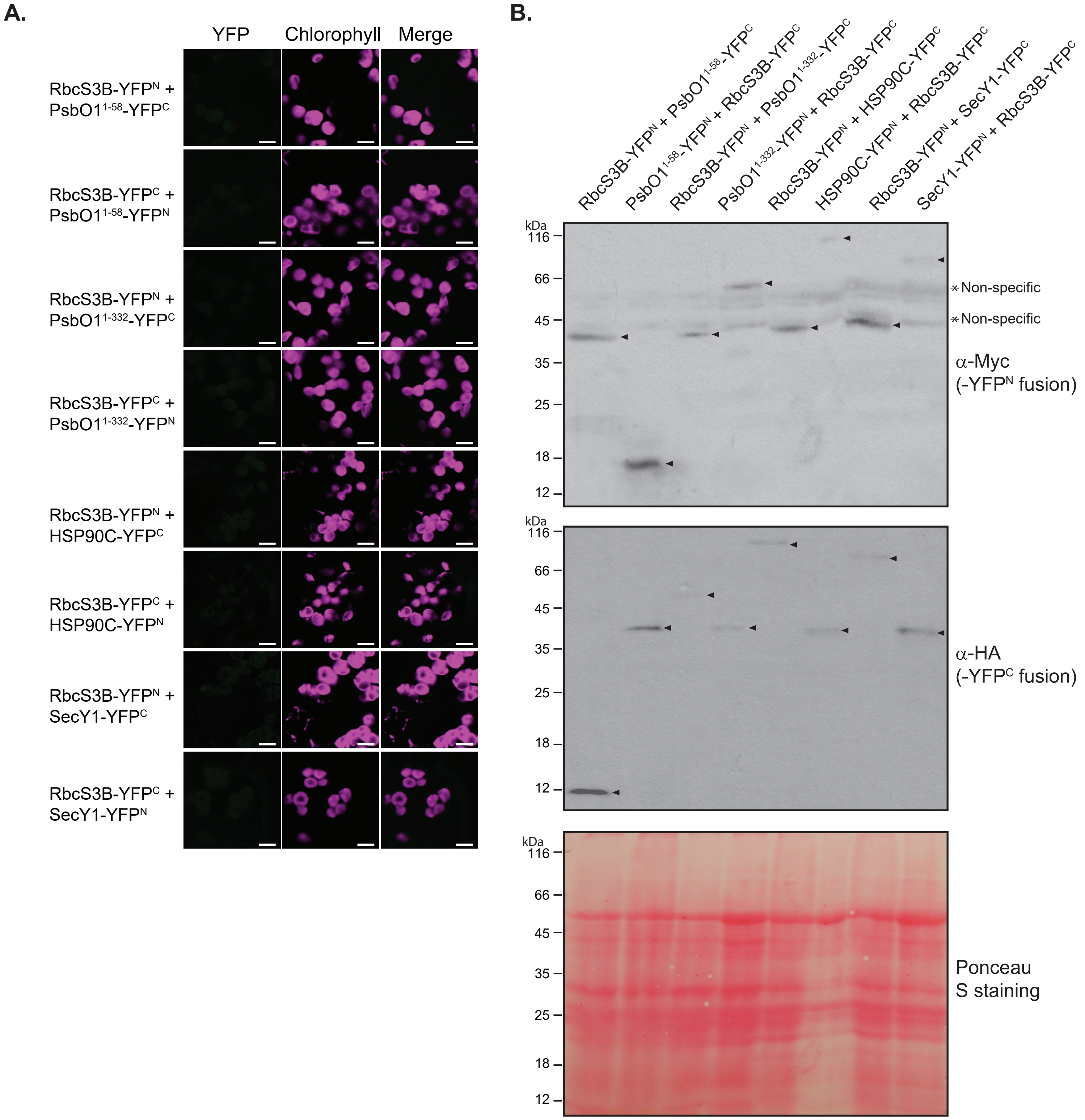

Figure S9. Bifluorescence complementation assay between HSP90C and RbcS3B. RbcS3B coding region was cloned into either pB7WGYN9 or pB7WGYC9 containing the nYFP/cYFP at their C-termini and then co-infiltrated with vectors containing HSP90C in *Agrobacterium tumefaciens*. **A.** fluorescence of YPF was observed three days after infiltration. No visible YFP signal was detected. **B.** Tobacco leaves transiently expressing split YFP fusion proteins were collected and 15 μg total soluble proteins were loaded to SDS-PAGE and immunoblotted with anti-Myc for -YFP^N fusion proteins and anti-HA for YFP^C fusion proteins, as the two halves are also tagged with Myc and HA epitopes. Arrows indicate transiently expressed fusion protein bands according expected fusion protein size, and Ponceau S staining membranes show equal loading.
